# Cerebrospinal fluid and dural accumulation of α-synuclein impairs CNS clearance in Parkinson’s disease

**DOI:** 10.1084/jem.20251245

**Published:** 2026-10-20

**Authors:** Taylor J. Stevenson, Luca Vinnell, Samuel J.C. McCullough, Daniel X. Flyger, Blake Highet, James A. Wiseman, Ben Watkin, Kate Hitpass Romero, Victoria F. Low, Amy M. Smith, Angela Wu, Dahna M. Fong, Natasha L. Grimsey, Birger V. Dieriks, Clinton P. Turner, Deborah Young, Maurice A. Curtis, Richard L.M. Faull, Mike Dragunow, Justin Rustenhoven

**Affiliations:** 1 Department of Pharmacology and Clinical Pharmacology, The University of Auckland, Auckland, New Zealand; 2 Centre for Brain Research, The University of Auckland, Auckland, New Zealand; 3 Department of Anatomy and Medical Imaging, The University of Auckland, Auckland, New Zealand; 4 LabPlus, Department of Anatomical Pathology, Te Whatu Ora, Auckland, New Zealand

## Abstract

Parkinson’s disease (PD) is characterized by the build-up of α-synuclein (α-syn) in the brain, where it drives neurodegeneration. Importantly, aggregated α-syn also accumulates in the cerebrospinal fluid (CSF) of PD patients, and while increasingly used for diagnosis, its pathological role remains poorly understood. Here, we demonstrate that α-syn accumulation in central nervous system (CNS) border tissues promotes CNS clearance deficits, as observed in PD patients. We find that aggregated α-syn accumulates not only in the CSF but also in the dura mater of individuals with PD, and, using acute and chronic mouse models, show that α-syn deposition in these sites induces immune alterations in CNS borders and disrupts CNS clearance. Mechanistically, we reveal that α-syn at CNS borders elevates CSF TNF and demonstrate that both TNF and α-syn aggregates themselves impair lymphatic endothelial cell function. These findings demonstrate a pathological role for α-syn in the CNS border tissues and position meningeal immunity as a contributor to CNS clearance deficits in PD pathogenesis.

## Teaser

Impaired waste clearance and chronic inflammation are emerging contributors to Parkinson’s disease pathogenesis. Here, Stevenson et al. show that aggregated α-synuclein at CNS borders promotes TNF-driven meningeal inflammation and impairs CNS clearance, identifying these tissues as potential therapeutic targets.

## Graphical Abstract

**Figure:**
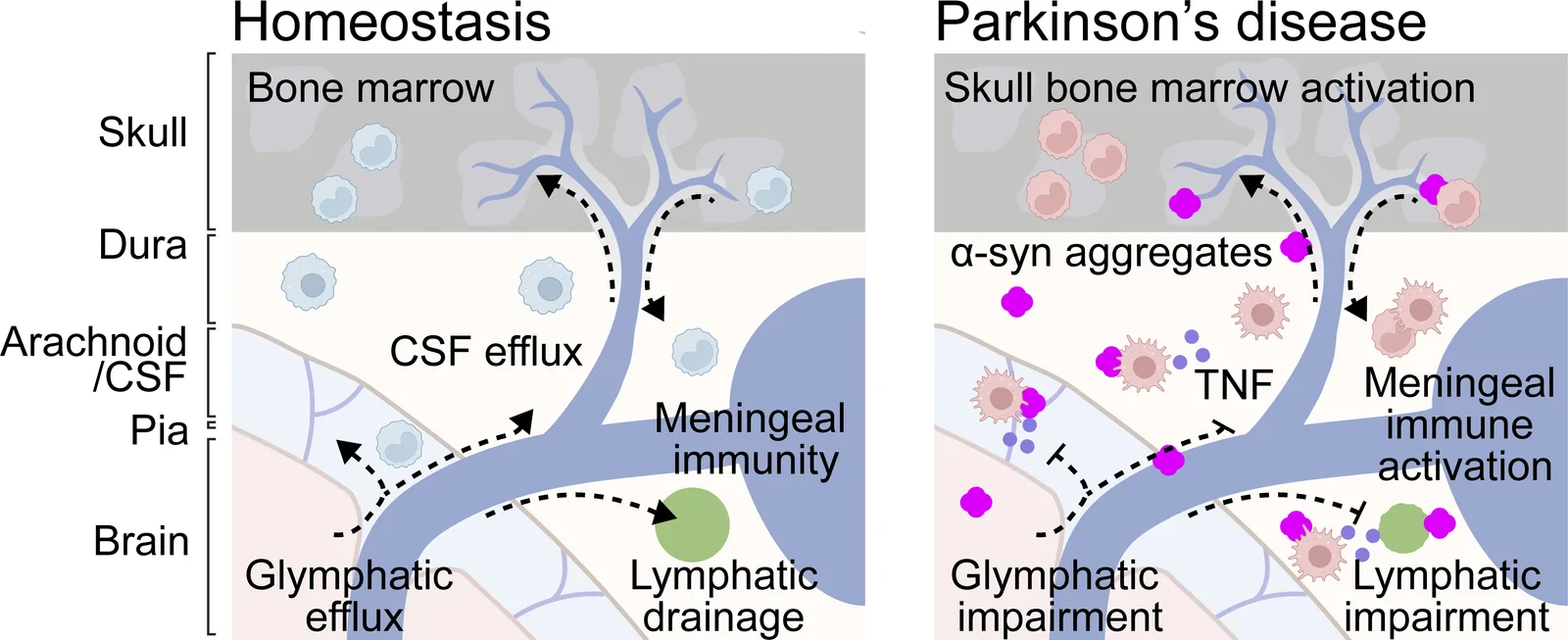

## Introduction

The central nervous system (CNS) relies on specialized clearance pathways to remove metabolic waste and maintain neurological health (Kim et al., 2025; Kipnis et al., 2025; Rustenhoven and Kipnis, 2022). Through the glymphatic system, interstitial waste from the brain parenchyma is exchanged with the cerebrospinal fluid (CSF) and subsequently drained through meningeal and nasal lymphatics to the cervical LNs. These pathways converge at CNS border tissues, particularly the meninges and associated lymphatic structures, which function as sites of both waste clearance and immune surveillance (Louveau et al., 2018; Mazzitelli et al., 2023; Rustenhoven et al., 2021; Rustenhoven and Kipnis, 2022; Smyth et al., 2024). Dysfunctions in these clearance pathways have been implicated in aging (Hitpass Romero et al., 2025; Ma et al., 2017; Rustenhoven et al., 2023; Yoon et al., 2024), neurological diseases including Alzheimer’s and Parkinson’s disease (PD) (Da Mesquita et al., 2018; Da Mesquita and Rua, 2024; Ding et al., 2021; Han et al., 2025; Louveau et al., 2018; Rustenhoven et al., 2021; Zou et al., 2019), and traumatic brain injuries (Bolte et al., 2020). Notably, neuroimaging studies have demonstrated impaired meningeal lymphatic drainage in patients with idiopathic PD (Ding et al., 2021), alongside dysfunction of the glymphatic system (Rajai Firouzabadi et al., 2026), suggesting that CNS clearance pathways are broadly compromised and clinically relevant in PD.

PD is defined by the pathological accumulation of the misfolded protein α-synuclein (α-syn), which contributes to dopaminergic neuronal loss and progressive motor and non-motor symptoms (Bloem et al., 2021; Braak et al., 2003; Jankovic, 2008; Schapira et al., 2017). While α-syn pathology has been extensively studied in the brain parenchyma, α-syn aggregates have also been detected in peripheral compartments including the gut (Anis et al., 2022), blood (Sui et al., 2014), and liver (Reyes et al., 2021). Further, aggregated α-syn is present in the CSF (Liu et al., 2024; Pierobon Mays et al., 2024; Poggiolini et al., 2022; Schmitz et al., 2016; Si et al., 2022; Wiseman et al., 2025) of PD patients and its detection using real-time quaking-induced conversion (RT-QuIC) is being widely explored to assist in the diagnosis (Poggiolini et al., 2022; Schmitz et al., 2016; Wiseman et al., 2025). However, the biological consequences of extracellular α-syn within the CSF remain poorly understood. This is important because CNS borders are increasingly recognized as neuroimmune hubs containing both innate and adaptive immune cells that regulate surveillance and clearance (Cugurra et al., 2021; Drieu et al., 2022; Mazzitelli et al., 2023; Rustenhoven et al., 2021; Schonhoff et al., 2023; Van Hove et al., 2019). Given that α-syn can engage peripheral immune populations, extracellular α-syn at these interfaces may represent a persistent inflammatory stimulus that contributes to neurodegeneration (Grozdanov et al., 2019; Lindestam Arlehamn et al., 2020; Schonhoff et al., 2023). This is particularly relevant given that enhanced α-syn clearance is widely viewed as a therapeutic goal in PD (Alfaidi et al., 2025).

Meningeal lymphatic vessels mediate CNS protein clearance by draining CSF solutes, including α-syn, to the deep cervical LNs (dCLNs) (Rustenhoven and Kipnis, 2022). Disruption of this pathway worsens glymphatic function, promotes α-syn accumulation, glial activation, and motor deficits in transgenic PD models (Zou et al., 2019). In parallel, α-syn drainage to dCLNs can activate macrophages and promote peripheral inflammation, while CNS α-syn exposure elevates cytokines such as IFN-γ that impair meningeal lymphatic function (Ding et al., 2021; Liu et al., 2023; Rustenhoven et al., 2023). However, whether extracellular α-syn within the CSF directly acts on CNS border tissues to impair lymphatic clearance remains unclear.

Here, we investigated whether extracellular α-syn aggregates at CNS borders drive functional and immunological disruption of these compartments in PD. Using a combination of human postmortem tissue analyses, seeding assays, and both acute and chronic α-syn–driven in vivo mouse models, we identify the dura mater of the meninges as a key site of α-syn deposition and inflammation from CSF α-syn aggregates. We show that α-syn disrupts CSF drainage through meningeal lymphatic vessels, promotes innate immune cell activation, and fosters a proinflammatory cytokine milieu, including elevated TNF levels. Notably, our findings demonstrate that α-syn can impair human lymphatic endothelial cell (LEC) function in vitro, both directly and indirectly through TNF-mediated signalling, highlighting a dual mechanism of lymphatic dysfunction. Together, these results reveal that α-syn accumulation at CNS interfaces is not merely a bystander but a key driver of lymphatic failure and neuroinflammation, positioning CNS border tissues as novel and actionable therapeutic targets in PD.

## Results and discussion

**Figure 1.**
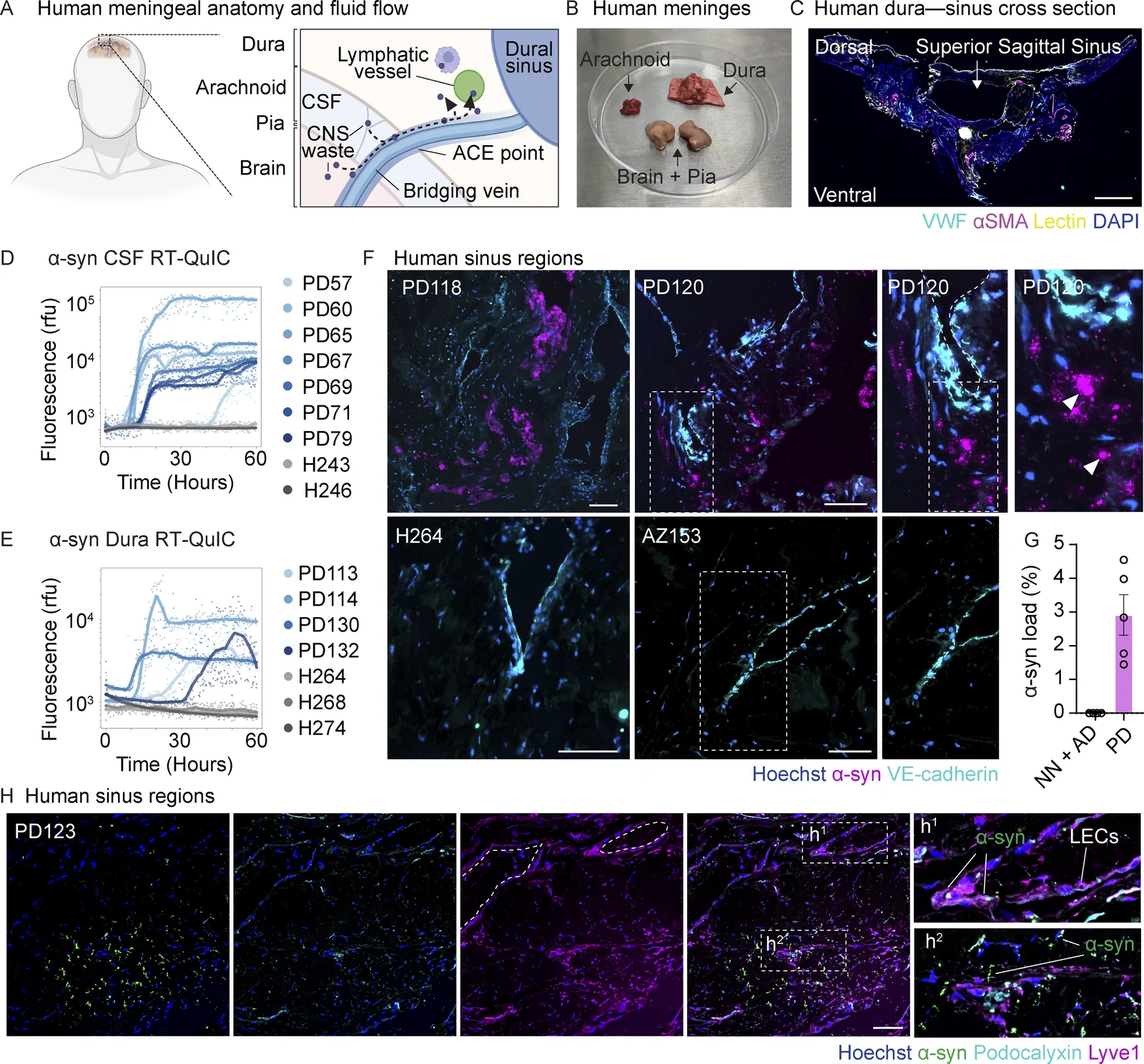
**α-syn aggregates are present in postmortem human dura mater and CSF in PD. (A)** Schematic illustration of human meningeal anatomy highlighting lymphatic vessels and CSF flow pathways at CNS borders. **(B)** Representative images of human brain and the three layers of the meninges, illustrating tissue samples collected. **(C)** Immunohistochemical staining of human dura mater showing the anatomy of the superior sagittal sinus. Scale bar = 2 mm. **(D and E)** Detection of α-syn seeding activity by RT-QuIC in (D) CSF (*n* = 2 neurologically normal [H], *n* = 7 [PD]) and (E) fresh-frozen dura mater (*n* = 3 neurologically normal [H], *n* = 4 [PD]) samples from individuals with PD and neurologically normal controls. **(F)** Immunohistochemical analysis of paraffin-embedded postmortem dura sections in the superior sagittal sinus, demonstrating discrete α-syn–positive inclusions (white arrowheads) in PD cases but not in neurologically normal (H) or Alzheimer’s disease (AZ) control tissues. Scale bar = 50 µm. **(G)** Quantification of α-syn pathology in dura mater from PD cases (*n* = 5) compared with control tissue (*n* = 4; two neurologically normal and two Alzheimer’s disease cases), expressed as percentage area of α-syn immunoreactivity. **(H)** Immunohistochemical analysis of paraffin-embedded postmortem dura sections in the superior sagittal sinus, demonstrating discrete intra/extracellular α-syn–positive inclusions in PD dura near Lyve1^+^ vessels. Scale bar = 100 µm.

The accumulation of aggregated α-syn is well established in the CSF of PD patients, but the functional consequences of this remain unclear (Bloem et al., 2021; Wiseman et al., 2025). Recent studies have identified anatomical routes linking the CSF with CNS border tissues, including the dura mater and skull bone marrow, suggesting these sites may be directly exposed to CSF-borne α-syn aggregates (Cugurra et al., 2021; Mazzitelli et al., 2023; Smyth et al., 2024). As meningeal lymphatic vessels are enriched around the dural sinuses, and arachnoid cuff exit (ACE) points provide routes for CSF access adjacent to these regions, dural tissue was specifically sampled from these areas (Jacob et al., 2022; Louveau et al., 2015; Smyth et al., 2024) (Fig. 1, A–C). We first investigated whether aggregated α-syn is found in the dura mater in human PD. Using postmortem CSF and sinus-associated dura mater from individuals with PD and neurologically normal controls (Table S1), we detected robust seeding activity in both CSF and fresh-frozen dura tissue from PD cases by RT-QuIC, while no seeding activity was observed in neurologically normal controls (Schmitz et al., 2016; Wiseman et al., 2025) (Fig. 1, D and E). Immunohistochemical analysis of paraffin-embedded dura around the superior sagittal sinus further revealed discrete α-syn–positive inclusions in PD cases, but not in neurologically normal or Alzheimer’s disease control tissue (Fig. 1, F and G). Both intracellular and extracellular α-syn aggregates were also closely associated with Lyve1^+^ vessels that were weakly podocalyxin^+^, revealing localization near dural lymphatic-associated vascular structures (Testa et al., 2009) (Fig. 1 H). These findings demonstrate that aggregated α-syn is not confined to the brain parenchyma or CSF in PD, but also accumulates in the dura mater, a key CNS border tissue involved in CSF drainage, immune surveillance, and waste clearance.

**Figure 2.**
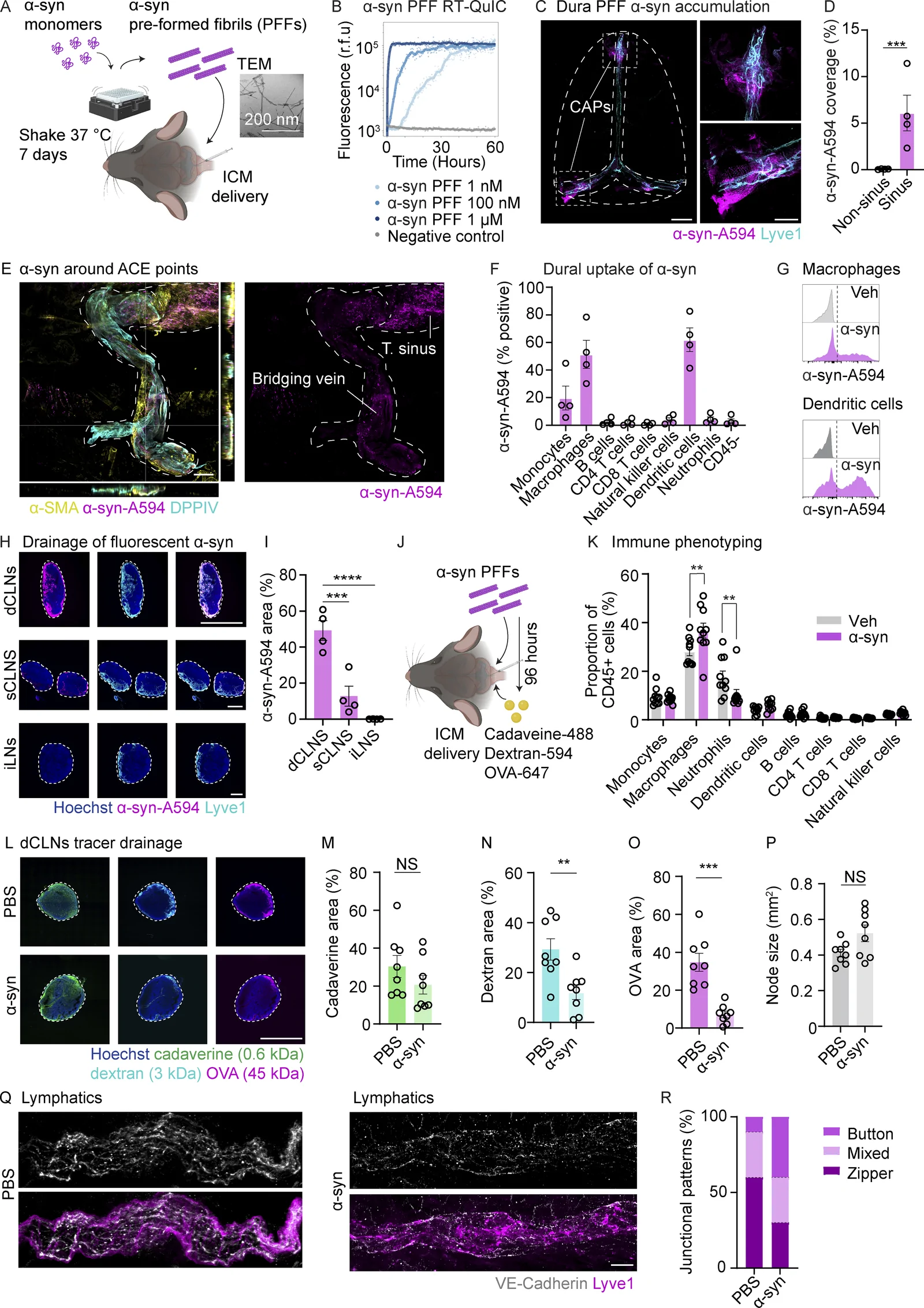
**Acute exposure to α-syn PFFs leads to accumulation at CNS borders, impaired lymphatic drainage, and immune activation. (A)** Schematic of the experimental design illustrating ICM injection of α-syn PFFs into 2-mo-old WT C57BL/6 mice and electron microscopy of the fibrillar structure of α-syn PFFs prior to injection. **(B)** Seeding competence of α-syn PFFs confirmed by RT-QuIC assay, demonstrating aggregation kinetics comparable with α-syn species detected in human dura and CSF. **(C–E)** Detection of fluorescent α-syn accumulation in CNS border tissues 24 h after injection of 2 μl of 100 µM PFFs, including (C and D) dura mater (*n* = 4 PBS mice, *n* = 4 α-syn PFF mice, ***P < 0.001 (Student's *t* test). Scale bar = 3,000 µm, left overview; scale bar = 200 µm, right magnification) and (E) ACE points (T. sinus = transverse sinus). Scale bar = 100 µm. **(F and G)** Flow cytometric analysis of fluorescent α-syn-594 PFF uptake by dura immune cells, including monocytes, macrophages, and dendritic cells after 24 h (*n* = 4 α-syn PFF mice). **(H)** Presence of fluorescent α-syn in dCLNs, superficial cervical LNs (sCLNs), but not inguinal LNs (iLNs) 24 h after ICM injection (*n* = 4 α-syn PFF mice). Scale bar = 500 µm. **(I)** Quantification of fluorescent α-syn in dCLNs, sCLNs and iLNs (*n* = 4 mice). ***P < 0.001, ****P < 0.0001 (one-way ANOVA with Tukey’s multiple-comparisons test). **(J)** Schematic of functional drainage experiment. α-syn PFFs were administered via ICM injection, followed 96 h later by injection of a fluorescent tracer cocktail including cadaverine-488 (0.6 kDa), dextran-594 (3 kDa), and OVA-647 (45 kDa) for lymphatic drainage assessment. **(K)** Spectral flow cytometry analysis of the dura 96 h after PFF exposure (data pooled from two independent experiments, *n* = 10 vehicle mice, *n* = 10 α-syn PFF mice). **P < 0.01 (two-way ANOVA with Sidak’s post hoc test). **(L–O)** Lymphatic drainage assessment to dCLNs of cadaverine-488, dextran-594, and OVA-647 96 h after PFF injection compared with controls (data pooled from two independent experiments, *n* = 8 vehicle mice, *n* = 8 α-syn PFF mice). NS P > 0.05, **P < 0.01, ***P < 0.001 (Student’s *t* test). Scale bar = 500 µm. **(P)** Quantification of dCLN node size between control and α-syn PFF-injected mice (data pooled from two independent experiments, *n* = 8 vehicle mice, *n* = 8 α-syn PFF mice). NS P > 0.05 (Student’s *t* test). **(Q and R)** Quantification of lymphatic junction structure in dura around CAPs comparing button, mixed, or zipper-like junctions (*n* = 5 vehicle mice, *n* = 5 α-syn PFF mice). Scale bar = 20 µm.

**Figure S1.**
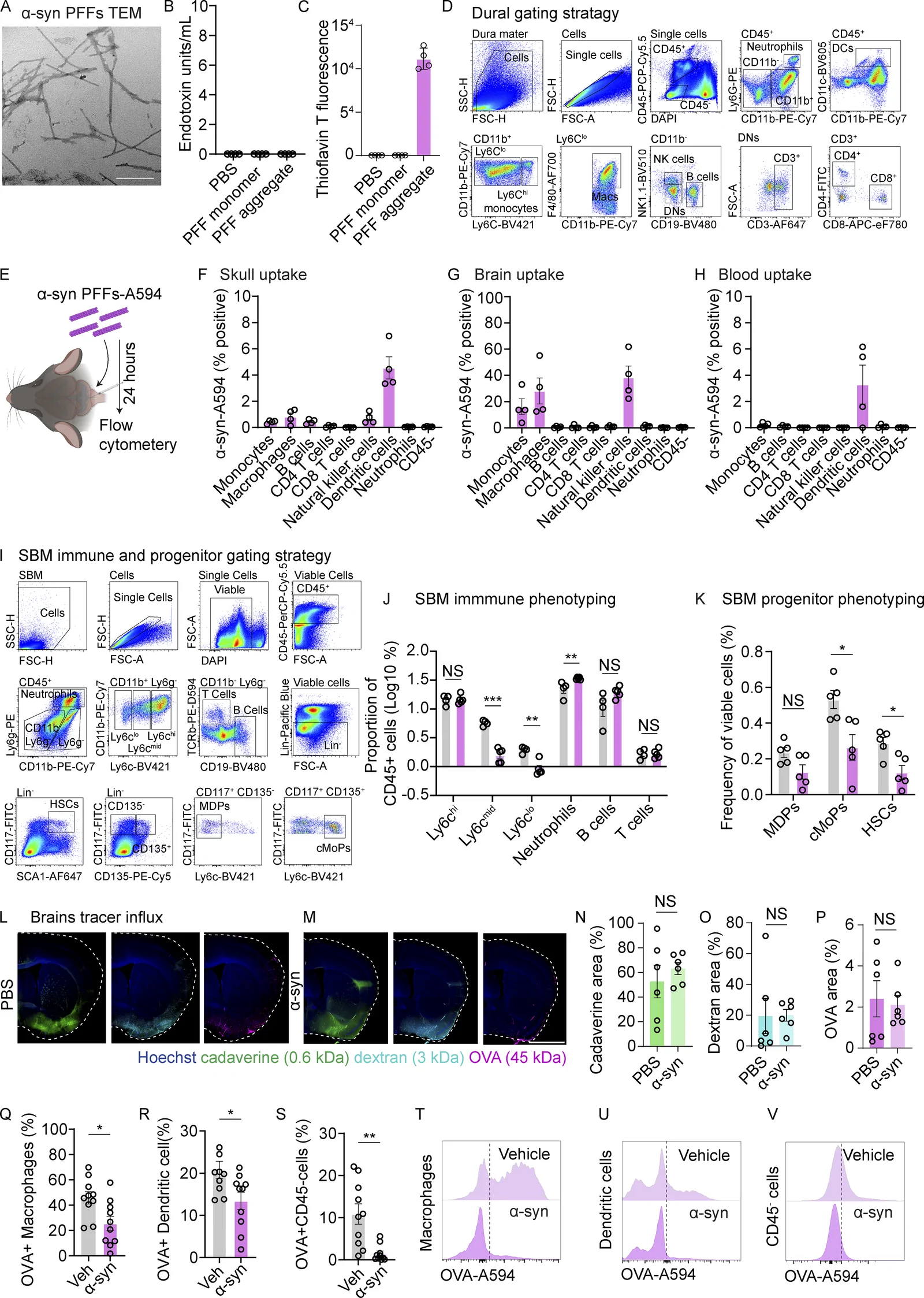
**Validation of α-syn PFFs and assessment of immune responses, α-syn distribution, glymphatic influx, and dural tracer uptake following acute CSF exposure.** **(A)** Electron microscopy confirms the fibrillar structure of α-syn PFFs prior to injection. Scale bar = 200 nm. **(B)** Endotoxin testing of α-syn PFF preparations using the LAL assay. **(C)** Thioflavin T staining demonstrates β-pleated sheet formation following fibrillization of α-syn PFFs. **(D)** Flow cytometry strategy for the identification of dural immune cells. **(E)** Schematic of the experimental design displaying ICM injection of fluorescent α-syn PFFs. **(F–H)** Flow cytometry analysis of fluorescent PFF uptake 24 h after injection by immune cell populations in the (F) skull, (G) brain, and (H) blood (*n* = 4 α-syn PFF mice). **(I)** Flow cytometry gating strategy for identification of skull immune cell populations and gating strategy for identification of skull bone marrow progenitor cell populations. **(J)** Immune phenotyping of the proportion of CD45^+^ (Log_10_) cells of skull immune populations between vehicle and α-syn PFF CSF delivered after 96 h for Ly6c^hi^, Ly6c^mi^, Ly6c^lo^ monocytes, neutrophils, B cells, and T cells (*n* = 4 PBS mice, *n* = 4 α-syn PFF mice). NS P > 0.05, **P < 0.01, and ***P <0.001 (two-way ANOVA with Sidak’s post hoc test). **(K)** Phenotyping of the proportion of viable skull progenitor cells between vehicle and α-syn PFF CSF delivered after 96 h for MDPs, cMoPs, and HSCs (*n* = 4 PBS mice, *n* = 4 α-syn PFF mice). NS P > 0.05, *P < 0.05 (two-way ANOVA with Sidak’s post hoc test). **(L–P)** Glymphatic influx assessment to brain parenchyma of fluorescent tracer cocktail of (N) cadaverine-488, (O) dextran-594, and (P) OVA-647 96 h after α-syn PFF injection compared with controls (*n* = 5 PBS mice, *n* = 5 α-syn PFF mice). NS P > 0.05 (Student’s *t* test). Scale bar = 1,000 µm. **(Q)** Flow cytometry analysis of OVA uptake in macrophages 96 h after PFF ICM injection (data pooled from two independent experiments, *n* = 10 PBS mice, *n* = 10 α-syn PFF mice). *P < 0.05 (Student’s *t* test). **(R)** Flow cytometry analysis of OVA uptake in dendritic cells 96 h after PFF ICM injection (data pooled from two independent experiments, *n* = 10 PBS mice, *n* = 10 α-syn PFF mice). *P < 0.05 (Student’s *t* test). **(S)** Flow cytometry analysis of OVA uptake in CD45^−^ cells 96 h after PFF ICM injection (data pooled from two independent experiments, *n* = 10 PBS mice, *n* = 10 α-syn PFF mice). **P < 0.01 (Student’s *t* test). **(T–V)** Flow cytometry analysis of OVA uptake in macrophages, dendritic cells, and CD45^−^ in PFF-treated mice compared with vehicle.

To investigate how CSF-derived α-syn aggregates interact with CNS border tissues, we next developed an acute mouse model in which α-syn pre-formed fibrils (PFFs) (Patterson et al., 2019) were injected into the CSF via intra-cisterna magna (ICM) injection in 2-mo-old WT C57BL/6 mice (Fig. 2 A). The injected PFFs were fibrillar in structure, endotoxin-free, Thioflavin T-positive (Fig. S1, A–C), and seeding competent by RT-QuIC (Fig. 2 B), with aggregation kinetics resembling α-syn species detected in human CSF and dura (Fig. 1, D and E). Following ICM injection, fluorescent α-syn PFFs were detected in several CNS-associated tissues 24 h later. In the dura, α-syn PFF accumulation was largely confined to CSF access points (CAPs) surrounding the dural sinuses (Rustenhoven et al., 2021) (Fig. 2, C and D) and ACE points, as previously seen with amyloid beta in 5xFAD mouse models (Smyth et al., 2024; Smyth et al., 2026) (Fig. 2 E). Flow cytometry of dural tissue confirmed that fluorescent α-syn PFFs were taken up by multiple dural immune cell populations, including monocytes, macrophages, and dendritic cells (Fig. 2, F and G; and Fig. S1 D). α-Syn uptake was also observed in phagocytes in the brain and leptomeninges, with lower amounts being observed in the skull bone marrow and blood (Fig. S1, E–H). Fluorescent α-syn PFFs were also detected in the dCLNs, superficial cervical LN, but not inguinal LNs 24 h later, consistent with drainage through CNS lymphatic pathways (Chayama et al., 2026; Jin et al., 2025; Yoon et al., 2024) (Fig. 2, H and I). Together, these results demonstrate that CSF-delivered α-syn rapidly reaches multiple CNS and border-associated tissues, including the dura surrounding the dural sinuses and lymphatic vessels, where it is taken up by innate immune populations and drained to local LNs by dural lymphatic vessels.

Given that α-syn can drive innate immune responses within the brain (Allen Reish and Standaert, 2015), we next investigated whether CSF α-syn exposure altered immune responses at CNS borders. Spectral flow cytometry analysis of the dura following acute (96 h) α-syn PFF exposure revealed a significant increase in macrophages, relative to vehicle controls (Fig. 2, J and K). In parallel, immune profiling of the skull bone marrow showed alterations in innate immune populations, including increased neutrophils, alterations in Ly6c monocyte proportions, and expansion of bone marrow progenitor populations including hematopoietic stem cells and common monocyte progenitors (Fig. S1, I–K). These findings suggest that CSF α-syn exposure induces a coordinated innate immune response across the dura and adjacent skull bone marrow compartments that supply myeloid cells to the dura (Cugurra et al., 2021).

As dural immune responses have previously been shown to impair CNS clearance pathways (Rustenhoven et al., 2023), we next assessed whether α-syn exposure altered glymphatic and lymphatic function. Mice exposed to α-syn PFFs for 96 h showed significantly reduced drainage of fluorescent tracers of varying sizes, including dextran (3 kDa) and OVA (45 kDa), but not cadaverine (0.6 kDa) to the dCLNs (Fig. 2, L–O). There were no significant changes in dCLNs node size (Fig. 2 P). In this acute model, there were no significant differences in the different-sized tracer coverage throughout the brain parenchyma, indicating no change in glymphatic influx (Fig. S1, L–P). Uptake of OVA by dural macrophages, dendritic cells, and CD45^−^ cells was also reduced, suggesting that α-syn exposure may alter CSF access to the dura, phagocyte uptake, or both (Fig. S1, Q–V). Additionally, we observed alterations in lymphatic vessel junction composition, with a greater shift towards button and mixed junctional patterns in the α-syn group compared with controls (Fig. 2, Q and R). As lymphatic endothelial junctions regulate vessel barrier integrity in a size-dependent manner, this junctional remodelling may reduce the ability of lymphatic vessels to retain and transport larger tracers to the dCLNs, leading to local leakage, retention, or inefficient downstream movement of macromolecules, while smaller solutes remain relatively preserved (Baluk et al., 2007; Jannaway and Scallan, 2021). Consistent with this, direct disruption of VE-cadherin in meningeal lymphatic vessels impairs CSF drainage of 45 kDa OVA to dCLNs, and altered meningeal lymphatic junctions are a feature of aging-associated lymphatic dysfunction (Ahn et al., 2019; Da Mesquita et al., 2018; Rustenhoven et al., 2023). Collectively, these data demonstrate that acute exposure to extracellular α-syn aggregates is sufficient to drive border immune activation, lymphatic junctional remodelling, and size-selective impairment of CNS clearance.

**Figure 3.**
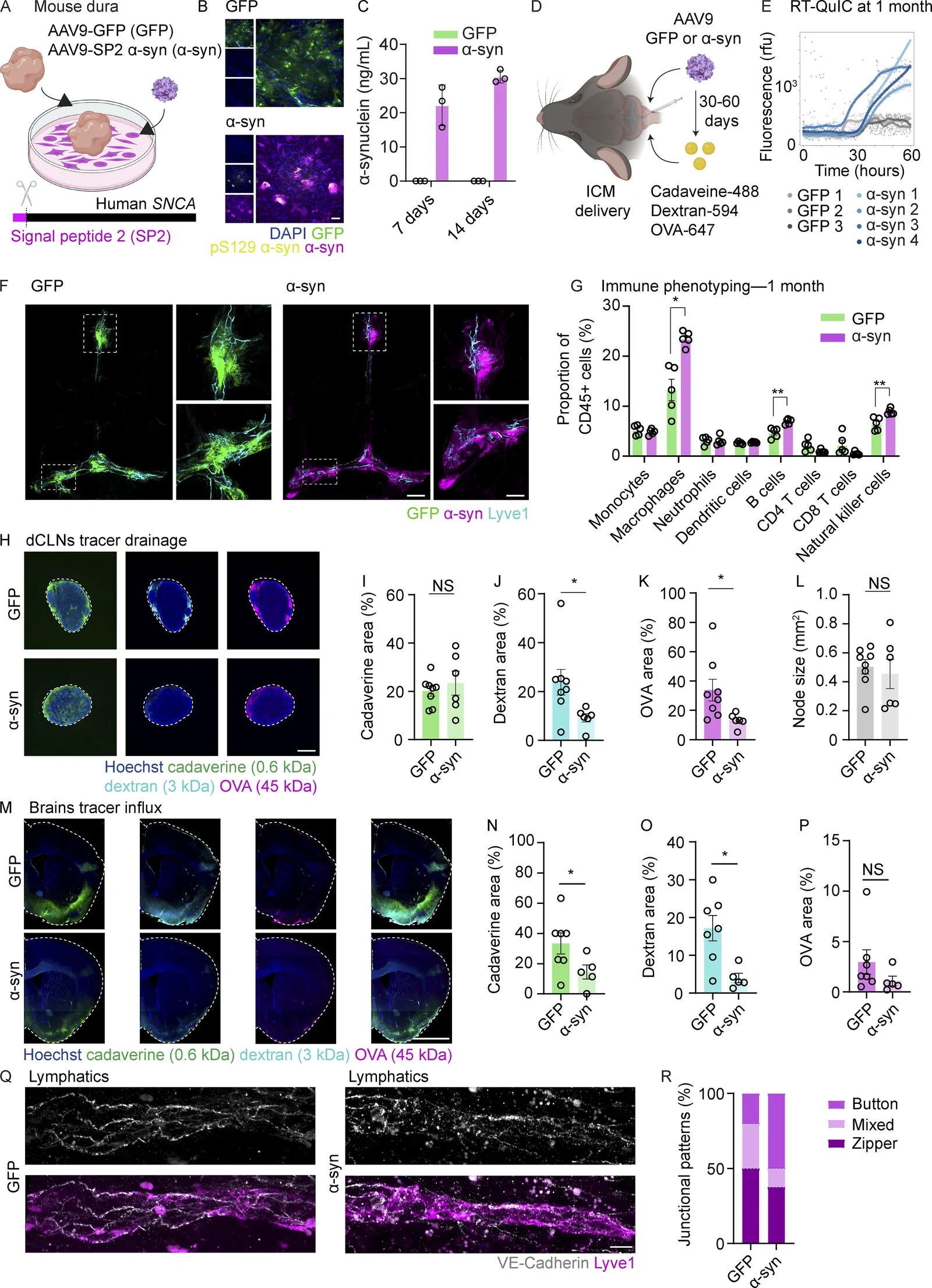
**Chronic CSF-derived α-syn exposure leads to sustained CNS border accumulation, impaired clearance, and immune activation. (A)** Schematic for the growth and transduction of mouse dural fibroblasts. **(B)** Immunocytochemistry of mouse fibroblasts 1 wk after transduction with AAV9 GFP (1 × 10^11^ GC/ml) and AAV9 encoding SP2-tagged α-syn (1 × 10^11^ GC/ml). Scale bar = 50 µm. **(C)** Quantification of secreted α-syn by ELISA after 7 and 14 days in mouse fibroblasts (*n* = 3 mice). **(D)** Schematic of experimental design showing ICM injection of AAV9-GFP or AAV9-SP2-α-syn. **(E)** Seeding-competent α-syn detected in CSF 1 mo after AAV delivery by RT-QuIC. **(F)** Immunohistochemistry of GFP and α-syn expression in CAPs 1 mo after AAV delivery (3 μl of 1 × 10^13^ GC/ml) in the dura. Scale bar = 3,000 µm, left overview; scale bar = 200 µm, right magnification. **(G)** Spectral flow cytometry of the dura 1 mo after CSF-delivered AAV9s (*n* = 5 AAV9-GFP mice, *n* = 5 AAV9-α-syn mice). *P < 0.05, **P < 0.01 (two-way ANOVA with Sidak’s post hoc test). **(H–K)** Lymphatic drainage assessment to dCLNs of fluorescent tracer cocktail of cadaverine-488 (0.6 kDa), dextran-594 (3 kDa), and OVA-647 (45 kDa) 1 mo after AAV9-α-syn injection compared with GFP controls (data pooled from two independent experiments, *n* = 8 AAV-GFP mice, *n* = 6 AAV9-α-syn mice). NS P > 0.05, *P < 0.05 (Student’s *t* test). Scale bar = 200 µm. **(L)** Quantification of dCLN node size between AAV9-GFP control and AAV9-α-syn–injected mice (data pooled from two independent experiments, *n* = 8 AAV9-GFP mice, *n* = 6 AAV9-α-syn mice). NS P > 0.05 (Student’s *t* test). **(M–P)** Glymphatic influx assessment to brain parenchyma of a fluorescent tracer cocktail of cadaverine-488, dextran-594, and OVA-647 1 mo after AAV9-α-syn injection compared with GFP controls (data pooled from two independent experiments, *n* = 7 AAV-GFP mice, *n* = 5 AAV9-α-syn mice). NS P > 0.05, *P < 0.05 (Student’s *t* test). Scale bar = 1,000 µm. **(Q and R)** Quantification of lymphatic junction structure in dura around CAPs 1 mo after AAV injection, comparing button, mixed, or zipper-like junctions (*n* = 5 AAV9-GFP mice, *n* = 5 AAV9-α-syn mice). Scale bar = 20 µm.

**Figure S2.**
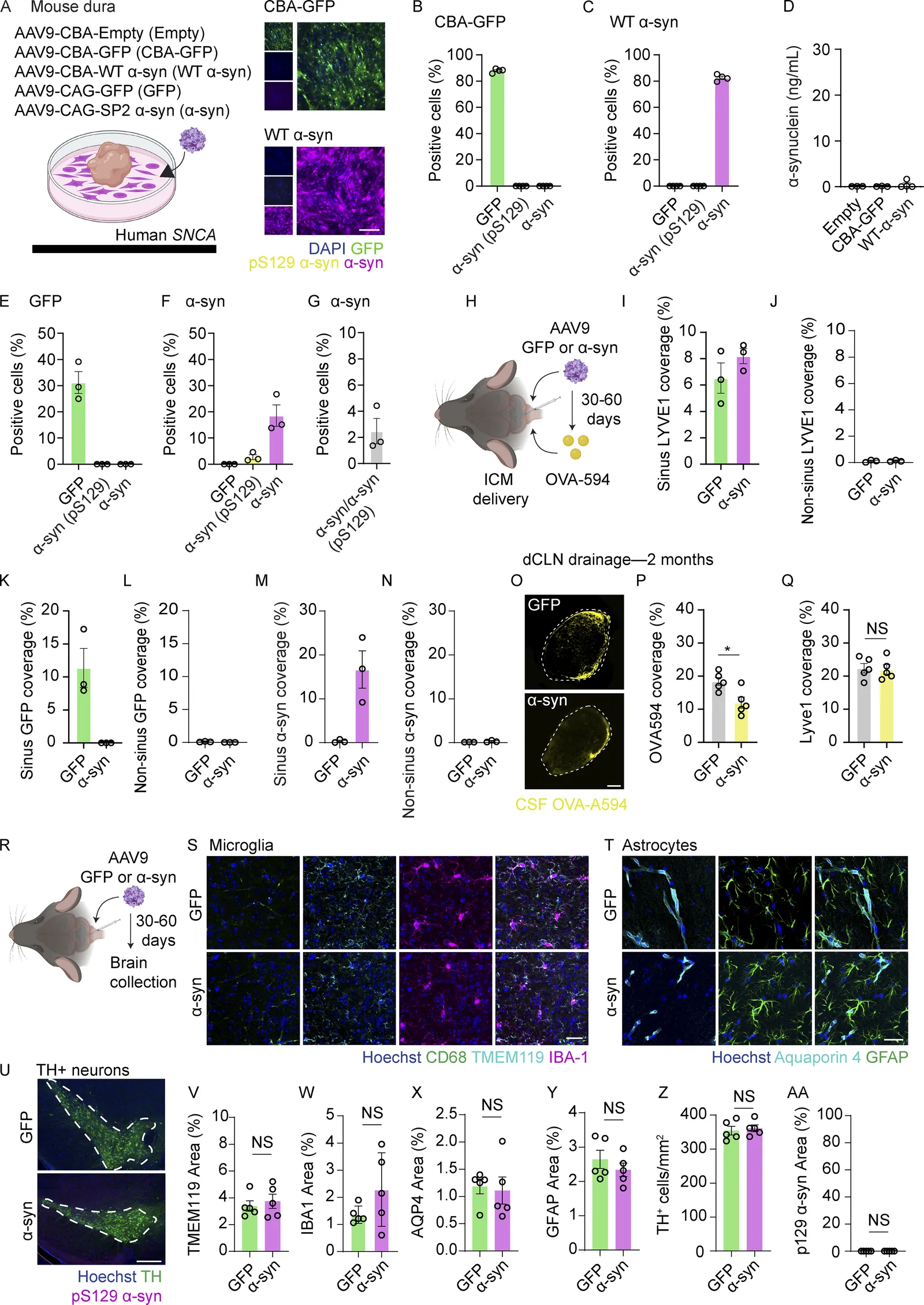
**Validation of AAV9-α-syn constructs and assessment of dural α-syn accumulation, lymphatic drainage, and brain parenchymal pathology following chronic CSF exposure** **. (A)** Immunocytochemistry of AAV9-CBA-GFP (1 × 10^11^ GC/ml) and AAV9-CBA-WT α-syn (1 × 10^11^ GC/ml) transduction of mouse fibroblasts after 1 wk of treatment. Scale bar = 200 µm. **(B)** Quantification of GFP expression in AAV9-CBA-GFP–treated mouse fibroblasts (*n =* 3 mice). **(C)** Quantification of α-syn and phospho-α-syn in AAV9-CBA-WT α-syn–treated mouse fibroblasts (*n* = 3 mice). **(D)** Quantification of secreted α-syn by ELISA after 7 and 14 days in mouse fibroblasts (*n =* 3 mice). **(E)** Quantification of GFP expression in AAV9-GFP–treated mouse fibroblasts (*n* = 3 mice). **(F)** Quantification of α-syn and phospho-α-syn in AAV9-α-syn–treated mouse fibroblasts (*n* = 3 mice). **(G)** Quantification of the ratio of α-syn compared with phospho-S129 α-syn antibody in mouse fibroblasts (*n* = 3 mice). **(H)** Schematic of experimental design showing ICM injection of AAV9-GFP or AAV9-SP2-α-syn. **(I–N)**. Quantification of Lyve1, GFP, and α-syn around the sinus and non-sinus regions in the dura 1 mo after AAV9-CAG-GFP and AAV9-CAG-SP2- α-syn (*n =* 3 mice). **(O–Q)** Lymphatic drainage assessment to dCLNs of (O) OVA594. Quantification of (P) OVA-594 drainage and (Q) Lyve1 expression in LNs taken 2 mo after AAV delivery (*n* = 5 AAV9-GFP mice, *n* = 4 AAV9-α-syn mice). NS P > 0.05, *P < 0.05 (Student’s *t* test). Scale bar = 100 µm. **(R)** Schematic of AAV9 mouse model. **(S)** Immunohistochemistry of microglia morphology in AAV9-GFP compared with AAV9-α-syn brain tissue. Scale bar = 20 µm. **(T)** Immunohistochemistry of astrocyte and aquaporin-4 morphology in AAV9-GFP compared with AAV9-α-syn brain tissue. Scale bar = 20 µm. **(U)** Immunohistochemistry of tyrosine-hydroxylase–positive neurons and phospho-α-syn staining in the substantia nigra in AAV9-GFP compared with AAV9-α-syn brain tissue. Scale bar = 200 µm. **(V)** Quantification of TMEM119 area in the cortex between AAV9-GFP and AAV9-α-syn brain tissue (*n* = 5 AAV9-GFP, *n* = 5 AAV9-α-syn). NS P > 0.05 (Student’s *t* test). **(W)** Quantification of IBA1 area in the cortex between AAV9-GFP and AAV9-α-syn brain tissue (*n* = 5 AAV9-GFP mice, *n* = 5 AAV9-α-syn mice). NS P > 0.05 (Student’s *t* test). **(X)** Quantification of Aquaporin-4 area in the cortex between AAV9-GFP and AAV9-α-syn brain tissue (*n* = 5 AAV9-GFP mice, *n* = 5 AAV9-α-syn mice). NS P > 0.05 (Student’s *t* test). **(Y)** Quantification of GFAP area in the cortex between AAV9-GFP and AAV9-α-syn brain tissue (*n* = 5 AAV9-GFP mice, *n* = 5 AAV9-α-syn mice). NS P > 0.05 (Student’s *t* test). **(Z)** Quantification of TH^+^ neurons in the substantia nigra between AAV9-GFP and AAV9-α-syn brain tissue (*n* = 5 AAV9-GFP mice, *n* = 5 AAV9-α-syn mice). NS P > 0.05 (Student’s *t* test). **(AA)** Quantification of phosphor-α-syn area in the substantia nigra between AAV9-GFP and AAV9-α-syn brain tissue (*n* = 5 AAV9-GFP mice, *n* = 5 AAV9-α-syn mice). NS P > 0.05 (Student’s *t* test).

Although the α-syn PFF model recapitulates α-syn accumulation at the dura mater and other CNS border tissues, it represents an acute exposure paradigm, and the persistence of PFF-derived α-syn over time remains unclear. To model the sustained effects of extracellular α-syn on CNS border tissues, we generated an AAV9 vector encoding human α-syn fused to an SP2 secretion signal under the control of the CAG promoter (Prymaczok et al., 2024), enabling continuous secretion of α-syn into the CSF and surrounding border tissues. In vitro validation using mouse meningeal fibroblasts confirmed robust α-syn expression and secretion into the culture medium (Fig. 3, A–C). In contrast, fibroblasts transduced with non-secreted WT α-syn (where the SP2 tag is not present) showed intracellular expression without detectable secretion by ELISA (Fig. S2, A–G).

Following ICM delivery of AAV9 vectors in vivo (Fig. 3 D), seeding-competent α-syn was detected in the CSF 1 mo later by RT-QuIC (Fig. 3 E). Immunohistochemical analysis demonstrated α-syn accumulation in the dura, particularly around CAPs and Lyve1^+^ lymphatic vessels, with no expression in non-sinus regions (Fig. 3 F and Fig. S2, H–N). Consistent with the acute PFF model, chronic α-syn exposure increased dural macrophage numbers via flow cytometric analysis (Fig. 3 G), indicative of sustained innate immune activation at CNS borders. Functionally, AAV-mediated α-syn secretion similarly impaired lymphatic drainage with significant reductions in dextran (3 kDa) and OVA (45 kDa) but not cadaverine (0.6 kDa) fluorescent tracers to the dCLNs at 1 mo (Fig. 3, H–K). This phenotype persisted through to 2 mo after ICM delivery of AAV9 vectors, showing a significant reduction in drainage of OVA to the dCLNs in α-syn mice (Fig. S2, O–Q). Similar to the acute PFF model, there were no significant differences in dCLN node size (Fig. 3 L). In this chronic model of α-syn exposure, there were significant differences in the different-sized tracer coverage throughout the brain parenchyma, with a significant reduction in cadaverine (0.6 kDa) and dextran (3 kDa) but not OVA (45 kDa) (Fig. 3, M–P), suggesting alterations to glymphatic influx. Similar to the acute model, we observed alterations in lymphatic vessel junction composition, with a greater shift towards button junctional patterns in the α-syn group compared with GFP control (Fig. 3, Q and R). These findings demonstrate that chronic extracellular α-syn expression at CNS borders is sufficient to induce persistent immune activation and long-lasting impairment of meningeal lymphatic drainage.

**Figure S3.**
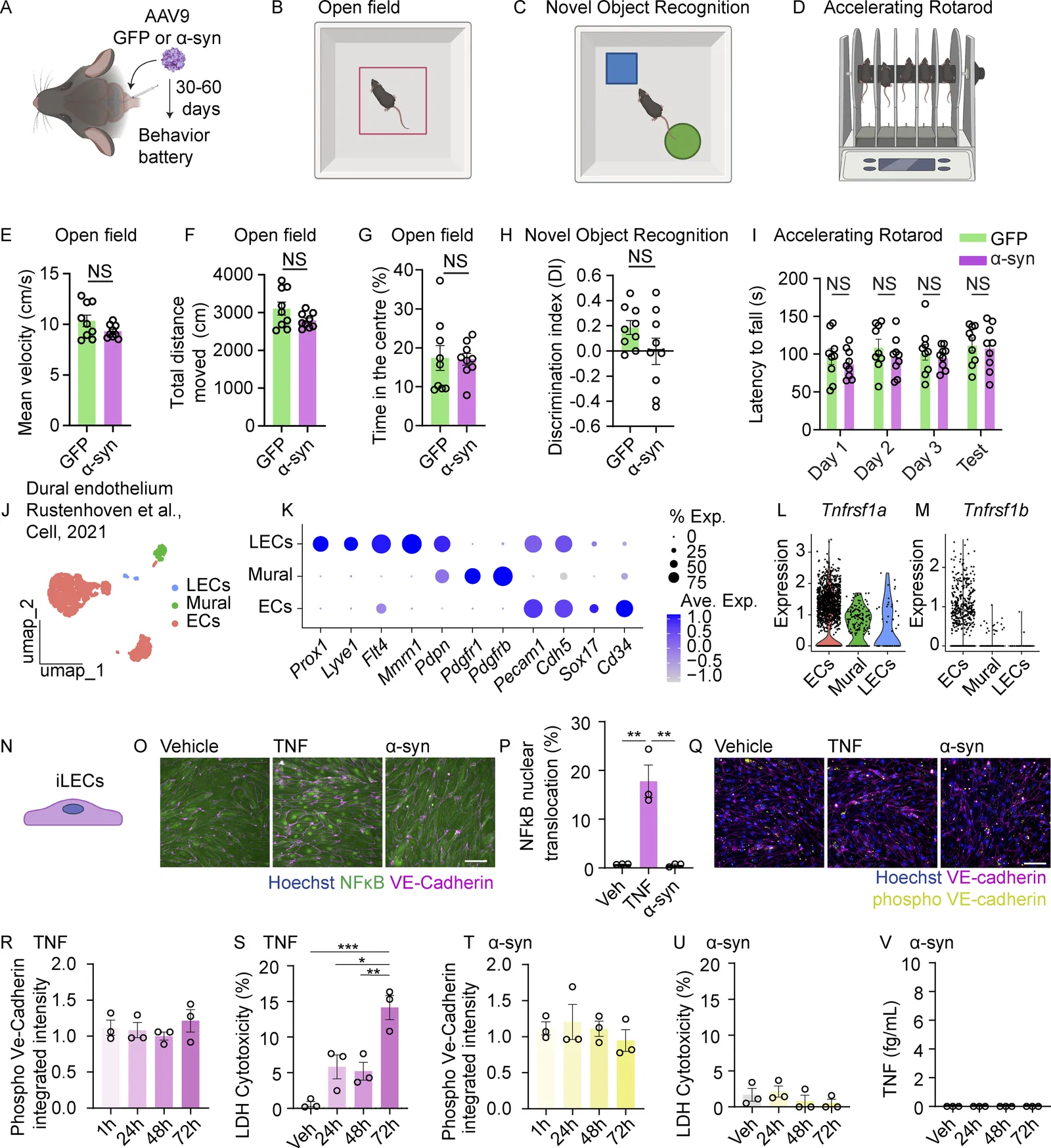
**Behavioral assessment of AAV9-α-syn mice and analysis of TNF receptor expression, signaling, cytotoxicity, and TNF release in LECs** **. (A)** Schematic of AAV9 mouse model. **(B)** Schematic of the open field test. **(C)** Schematic of the novel object recognition test. **(D)** Schematic of the accelerating rotarod test. **(E)** Quantification of mean velocity (cm/s) in a 40 × 40 cm arena between AAV9-GFP and AAV9-α-syn mice 1 mo after injection (data pooled from two independent experiments, *n* = 9 AAV9-GFP mice, *n* = 9 AAV9-α-syn mice). NS P > 0.05 (Student’s *t* test). **(F)** Quantification of total distance moved (cm) in a 40 × 40 cm arena between AAV9-GFP and AAV9-α-syn mice 1 mo after injection (data pooled from two independent experiments, *n* = 9 AAV9-GFP mice, *n* = 9 AAV9-α-syn mice). NS P > 0.05 (Student’s *t* test). **(G)** Quantification of time spent in the center (%) in a 40 × 40 cm arena between AAV9-GFP and AAV9-α-syn mice 1 mo after injection (data pooled from two independent experiments, *n* = 9 AAV9-GFP mice, *n* = 9 AAV9-α-syn mice). NS P > 0.05 (Student’s *t* test). **(H)** Discrimination index of the novel object recognition task between AAV9-GFP and AAV9-α-syn mice 1 mo after injection (data pooled from two independent experiments, *n* = 9 AAV9-GFP mice, *n* = 9 AAV9-α-syn mice). NS P > 0.05 (Student’s *t* test). **(I)** Accelerating rotarod investigating the latency to fall (s) between AAV9-GFP and AAV9-α-syn mice 1 mo after injection (data pooled from two independent experiments, *n* = 9 AAV9-GFP mice, *n* = 9 AAV9-α-syn mice). NS P > 0.05 (Student’s *t* test). **(J–M)** Reanalysis of a published scRNA-seq dataset from the dural endothelium (Rustenhoven et al., 2021). **(J)** UMAP projections from scRNA-seq analysis of endothelial cells from the dura of WT mice. **(K)** Dot plot for phenotyping of endothelial cells identifying the LEC cluster as LECs. **(L and M)** Violin plots displaying *Tnfrsf1a* and *Tnfrsf1b* receptor mRNA expression in LECs. **(N and O)** Immunocytochemistry of iLECs treated for 1 h with TNF (10 ng/ml) or α-syn (500 nM) for NF-κB translocation. Scale bar = 20 µm. **(P)** Quantification of NF-κB translocation in iLECs after 1 h of treatment with TNF (10 ng/ml) or α-syn (500 nM) (*n* = 3 independent iLEC differentiations from *n* = 3 iPSC lines). **P < 0.01 (one-way ANOVA with Tukey’s multiple-comparisons test). **(Q)** Immunocytochemistry of iLECs treated for 24 h with TNF (10 ng/ml) or α-syn (500 nM) for phospho-VE-Cadherin. Scale bar = 20 µm. **(R)** Quantification of phospho-VE-Cadherin in iLECs after 24-, 48-, or 72-h treatment with TNF (10 ng/ml) normalized to vehicle (*n* = 3 independent iLEC differentiations from *n* = 3 iPSC lines). NS P > 0.05 (one-way ANOVA with Tukey’s multiple-comparisons test). **(S)** LDH cytotoxicity assay in iLECs after 24-, 48-, or 72-h treatment with TNF (10 ng/ml) (*n* = 3 independent iLEC differentiations from *n* = 3 iPSC lines). *P < 0.05, **P < 0.01, ***P < 0.001 (one-way ANOVA with Tukey’s multiple-comparisons test). **(T)** Quantification of phospho-VE-Cadherin in iLECs after 24-, 48-, or 72-h treatment with α-syn (500 nM) normalized to vehicle (*n* = 3 independent iLEC differentiations from *n* = 3 iPSC lines). NS P > 0.05 (one-way ANOVA with Tukey’s multiple-comparisons test). **(U)** LDH cytotoxicity assay in iLECs after 24-, 48-, or 72-h treatment with α-syn (500 nM) (*n* = 3 independent iLEC differentiations from *n* = 3 iPSC lines). NS P > 0.05 (one-way ANOVA with Tukey’s multiple-comparisons test). **(V)** Quantification of TNF release from iLECs treated with α-syn (500 nM) for 24-, 48-, or 72-h using a cytometric bead array (*n* = 3 independent iLEC differentiations from *n* = 3 iPSC lines). NS P > 0.05 (one-way ANOVA with Tukey’s multiple-comparisons test).

To determine whether chronic CSF-derived α-syn exposure induced overt brain pathology or behavioral deficits, we next assessed parenchymal markers and performed behavioral testing following AAV9-SP2-α-syn delivery. Despite the clear immune and lymphatic alterations observed at CNS borders, we did not detect changes in microglial TMEM119, IBA1, or CD68 coverage, astrocytic GFAP, aquaporin-4 coverage, or TH-positive neuron numbers in the substantia nigra. Phosphorylated α-syn was also not detected within the brain parenchyma (Fig. S2, R–AA). Consistent with this, AAV9-SP2-α-syn mice showed no significant behavioral differences in the open field test, novel object recognition, or accelerating rotarod, indicating that locomotor activity, anxiety-like behavior, recognition memory, motor coordination, and motor learning were unchanged (Fig. S3, A–I). Together, these findings suggest that the lymphatic and immune changes observed at CNS borders occur without overt parenchymal α-syn accumulation, gliosis, aquaporin-4 alterations, dopaminergic neuron loss, or behavioral impairment and are therefore likely driven by direct effects of extracellular α-syn at CNS border tissues.

**Figure 4.**
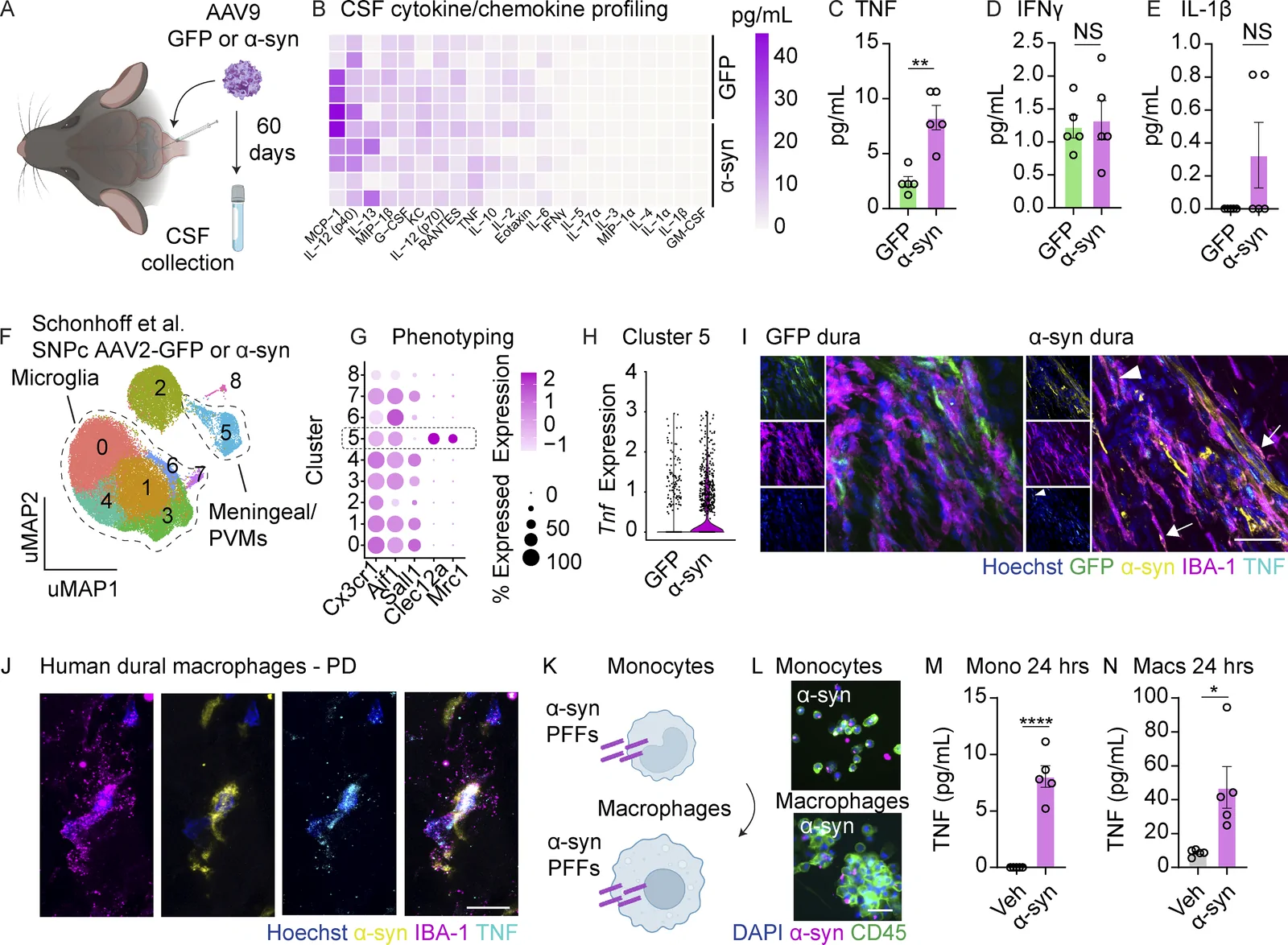
**Chronic α-syn exposure induces TNF-mediated inflammation in CNS border tissues. (A)** Schematic of experimental design showing ICM injection of AAV9-GFP or AAV9-SP2-α-syn and CSF collection 60 days later. **(B–E)** Luminex analysis of CSF collected 2 mo after AAV9-SP2-α-syn or AAV9-GFP (3 μl of 1 × 10^13^ GC/ml) showing (B) a heatmap and (C) significantly increased TNF, but not (D) IFNγ or (E) IL-1β (*n* = 5 AAV9-GFP mice, *n* = 5 AAV9-α-syn mice). NS P > 0.05, **P < 0.01 (Student’s *t* test). **(F–H)** Reanalysis of a published scRNA-seq dataset from a substantia nigra α-syn overexpression model (Schonhoff et al., 2023). **(F)** UMAP projections from scRNA-seq analysis of myeloid cells from the midbrains of mice 4 wk after AAV2-GFP and AAV2-α-syn injection into the substantia nigra pars compacta. **(G)** Dot plot for phenotyping of myeloid cells identifying cluster 5 as meningeal/PVMs. **(H)** Violin plots displaying *Tnf* induction from meningeal/PVMs in AAV-α-syn mice. **(I)** Immunohistochemistry of dura from AAV-GFP and AAV-α-syn mice 1 mo after injection showed TNF immunoreactivity in IBA1^+^ macrophages (white arrowhead) and phagocytosing α-syn aggregates (white arrows) in AAV-α-syn mice, but not in AAV-GFP controls. Scale bar = 50 µm. **(J)** Immunohistochemistry of postmortem human dura from a PD case exhibits TNF immunoreactivity in IBA1^+^ macrophages containing α-syn aggregates. Scale bar = 10 µm. **(K)** Schematic of THP-1 monocytes and macrophages treated with α-syn PFFs. **(L)** Immunocytochemistry of THP-1 monocytes and macrophages with phagocytosed α-syn PFFs (500 nM) after 24 h. Scale bar = 30 µm. **(M and N)** Quantification of TNF production using a cytometric bead array after treatment with α-syn PFFs (500 nM) in THP-1 monocytes and PMA-differentiated macrophages after 24 h (*n* = 5 independent passages) (Mono, monocytes; Macs, macrophages). *P < 0.01, ****P < 0.0001 (Student’s *t* test).

Given the sustained innate immune activation observed in both acute and chronic α-syn exposure models, we next sought to identify specific inflammatory mediators that may contribute to CNS border dysfunction. Multiplex (23-Plex) Luminex analysis of CSF collected 2 mo after AAV9-SP2-α-syn delivery revealed a significant increase in TNF protein levels compared with AAV9-GFP controls (Fig. 4, A–C). In contrast, other proinflammatory cytokines, including IFNγ and IL-1β, remained unchanged (Fig. 4, D and E). This suggests that extracellular α-syn exposure promotes a selective inflammatory cytokine response within the CSF.

To further interrogate the molecular signature of border macrophages following α-syn exposure, we reanalyzed a published single-cell RNA-sequencing (scRNA-seq) dataset profiling border-associated macrophages in a mouse model receiving AAV2-mediated α-syn overexpression in the substantia nigra (Schonhoff et al., 2023). This analysis also demonstrated the upregulation of *Tnf* expression within meningeal and perivascular macrophage clusters (Fig. 4, F–H). Immunostaining of dura from AAV9-GFP and AAV9-SP2-α-syn mice revealed increased TNF expression following α-syn exposure, with TNF localizing to IBA1^+^ macrophages containing phagocytosed α-syn, as well as non-IBA1^+^ cells (Fig. 4 I). Postmortem human dura samples from individuals with PD confirmed TNF immunoreactivity in IBA1^+^ macrophages in human PD dura containing α-syn aggregates, suggesting these cells may be a source of inflammatory cytokines in PD border tissues (Fig. 4 J). To determine whether macrophages were potential sources of TNF production, we exposed THP-1 monocytes and PMA-differentiated macrophages to α-syn PFFs (Fig. 4 K). Both produced TNF after 24 h of exposure (Fig. 4, L–N), consistent with previous findings that macrophages and microglia are a major source of TNF production (Alam et al., 2022; Béraud et al., 2013; Choi et al., 2020; Karampetsou et al., 2017; Liu et al., 2022). Together, these findings identify TNF as a conserved inflammatory mediator induced by α-syn exposure across mouse, human, and in vitro systems, and suggest that border-associated macrophages may be a likely source of this response.

**Figure 5.**
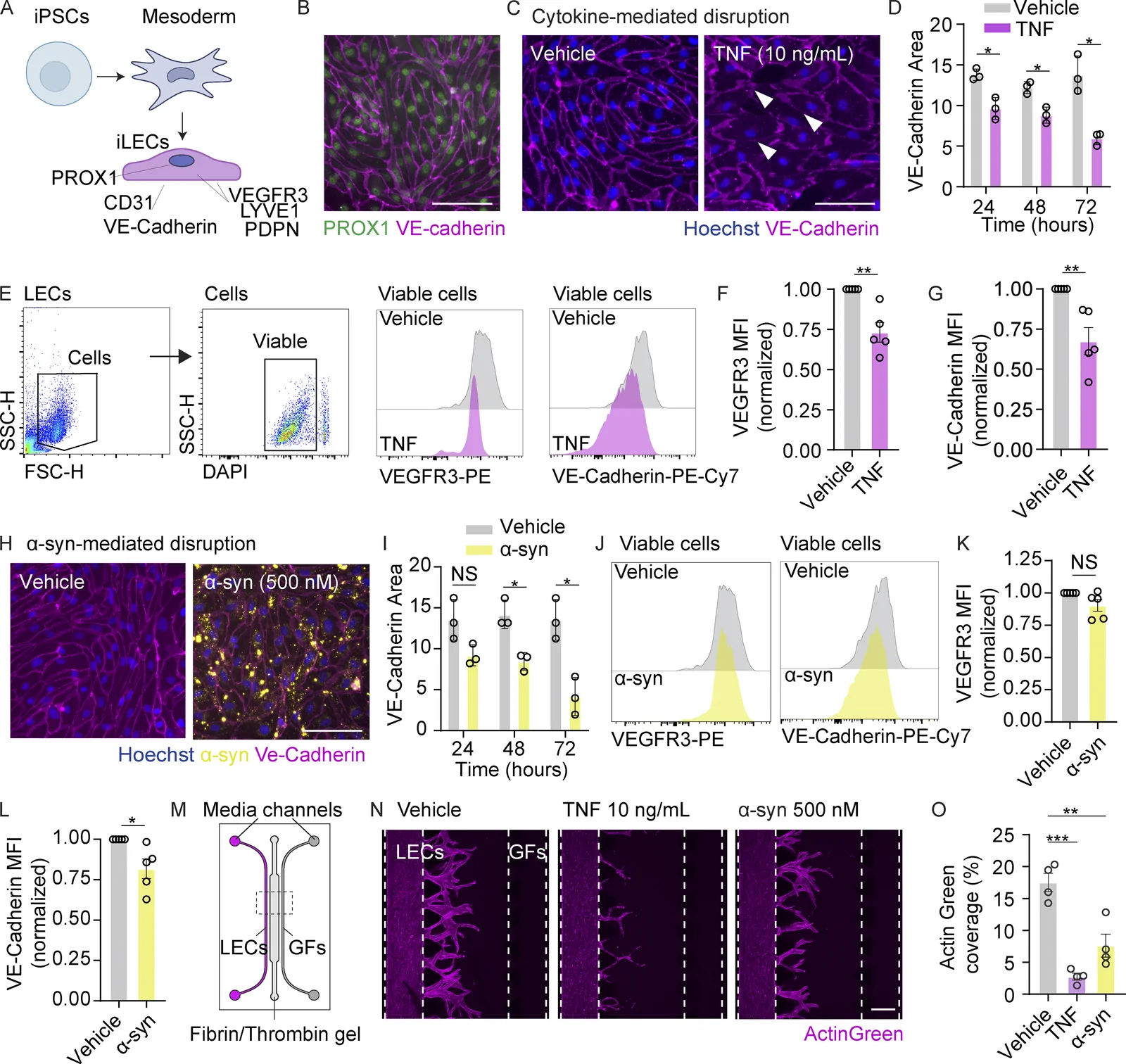
**TNF and α-syn disrupt lymphatic endothelial structure and sprouting capacity. (A)** Summary schematic for the development of iPSC-derived iLECs. iLECs express key lymphatic markers including PROX1 and VE-Cadherin. **(B)** Immunostaining confirms PROX1 and VE-cadherin expression in iLECs under baseline conditions. Scale bar = 50 µm. **(C)** Junctional disruption following 24-h TNF (10 ng/ml) treatment. **(D)** Quantification of VE-cadherin area over a 72-h time course demonstrates progressive loss of junctional integrity with TNF (10 ng/ml) exposure (*n* = 3 independent iLEC differentiations from *n* = 3 iPSC lines). *P < 0.05 compared with vehicle (two-way ANOVA with Sidak’s post hoc test). Scale bar = 50 µm. **(E–G)** Flow cytometry shows reduced surface expression of (F) VEGFR3 and (G) VE-Cadherin in TNF (10 ng/ml)-treated iLECs after 72 h (*n* = 3 independent iLEC differentiations from *n* = 3 iPSC lines). **P < 0.01 (Wilcoxon matched-pairs signed rank test). **(H and I)** Immunostaining of iLECs treated with α-syn PFFs (500 nM) shows VE-cadherin disruption over 72 h (*n* = 3 independent iLEC differentiations from *n* = 3 iPSC lines). *P < 0.05 compared with vehicle (two-way ANOVA with Sidak’s post hoc test). Scale bar = 50 µm. **(J–L)** Flow cytometry confirms α-syn PFFs (500 nM) reduce VE-Cadherin but not VEGFR3 expression after 72 h (*n* = 3 independent iLEC differentiations from *n* = 3 iPSC lines). NS P > 0.05, *P < 0.05 (Wilcoxon matched-pairs signed rank test). **(M)** Diagram of microfluidic lymphatic sprouting assay using fibrin/thrombin gels toward growth factors VEGF-C, Ang1, and HGF. **(N and O)** Quantification of iLECs labelled with ActinGreen coverage to measure sprouting in response to VEGF-C, Ang1, and HGF (100 ng/ml each) after TNF (10 ng/ml) and α-syn PFFs (500 nM) treatment on identX microfluidic chips after 6 days (*n* = 3 independent iLEC differentiations from *n* = 3 iPSC lines). **P <0.01, ***P <0.001 compared with vehicle (one-way ANOVA with Tukey’s multiple-comparisons test). Scale bar = 200 µm.

Proinflammatory cytokines such as IFNγ have previously been shown to be deleterious to meningeal lymphatic vessels and CSF drainage (Rustenhoven et al., 2023). We therefore investigated whether elevated TNF, or α-syn aggregates themselves, could directly impair LECs. To confirm that LECs can directly respond to TNF, we reanalyzed a published scRNA-seq dataset of dural endothelium in WT mice (Rustenhoven et al., 2021). LECs expressed *Tnfrsf1a*, with lower expression of *Tnfrsf1b*, supporting their capacity to directly respond to canonical TNF signalling (Fig. S3, J–M). Next, we treated human induced pluripotent stem cell (iPSC)-derived LECs (iLECs) (Hitpass Romero et al., 2025) with recombinant TNF or α-syn PFFs and assessed changes in barrier integrity and lymphatic signalling. These iLECs expressed canonical lymphatic markers, including PROX1, and displayed junctional VE-cadherin under baseline conditions, largely resembling collecting lymphatics (Fig. 5, A and B). Exposure to recombinant TNF induced NF-κB nuclear translocation after 1 h of treatment (Fig. S3, N–P) and disrupted VE-cadherin localization at cell–cell junctions, with progressive loss of VE-cadherin junctional area over 72 h (Fig. 5, C and D). Additionally, flow cytometry further demonstrated reduced surface VE-cadherin expression, accompanied by reduced VEGFR3 levels (Fig. 5, E–G), a receptor essential for lymphangiogenic signalling and vessel homeostasis (Antila et al., 2017). TNF treatment did not markedly alter phospho-VE-cadherin expression (Fig. S3, Q and R), while TNF induced some cytotoxicity (Fig. S3 S). As disruption of VE-cadherin in lymphatics results in lymphatic hyperpermeability and impaired CSF drainage, these data suggest that TNF may directly contribute to meningeal lymphatic dysfunction.

As our in vivo data suggested that TNF elevation follows extracellular α-syn accumulation, we next examined whether α-syn itself could impair LECs. Treatment of iLECs with recombinant α-syn PFFs also induced junctional disruption, with immunostaining showing a significant decrease in VE-cadherin area (Fig. 5, H and I), but with minimal NF-κB nuclear translocation (Fig. S3, O and P). Flow cytometry confirmed a significant reduction in VE-cadherin surface levels, while VEGFR3 expression remained unchanged (Fig. 5, J–L). Similar to TNF treatment, α-syn PFFs did not significantly alter phospho-VE-cadherin expression (Fig. S3 T). In contrast, α-syn PFFs did not induce significant cell cytotoxicity (Fig. S3 U), indicating a partially overlapping, but distinct, mode of lymphatic dysfunction compared with TNF. α-syn PFFs also do not induce TNF release by iLECs themselves (Fig. S3 V).

Lastly, to assess functional consequences on lymphangiogenesis, we employed a 3D microfluidic lymphatic sprouting assay in which iLECs form lumenized vessels within a fibrin/thrombin matrix (Hitpass Romero et al., 2025; Rustenhoven et al., 2023; Serrano et al., 2024) (Fig. 5 M). Under stimulation with lymphangiogenic cues (VEGF-C, Ang1, and HGF), vehicle-treated iLECs showed robust sprouting responses. In contrast, treatment with either TNF or α-syn significantly impaired lymphatic sprouting, resulting in reduced vessel extension and branching (Fig. 5, N and O).

Collectively, these findings demonstrate that both TNF and α-syn exert direct deleterious effects on LECs by disrupting junctional integrity, downregulating key surface receptors, and impairing lymphangiogenesis. These results support a mechanism whereby sustained inflammation and pathological α-syn burden at CNS borders contribute to dysfunction of meningeal lymphatic clearance pathways in PD.

Together, our data demonstrate that extracellular α-syn accumulation at CNS borders is not simply a downstream marker of PD pathology but can actively disrupt CNS clearance. We show that seeding-competent α-syn is present in both the CSF and dura mater of PD patients and that CSF exposure to α-syn in mice leads to accumulation around dural CAPs and lymphatic structures, uptake by border-associated phagocytes, innate immune activation, and impaired glymphatic and lymphatic clearance (Braak et al., 2003; Louveau et al., 2015; Malek et al., 2014; Mazzitelli et al., 2023; Rustenhoven et al., 2021; Smyth et al., 2024; Wiseman et al., 2025). Meningeal lymphatic dysfunction has previously been observed in PD patients and α-syn–based animal models, yet the mechanisms driving this impairment have remained unclear (Ding et al., 2021; Zou et al., 2019). Our findings suggest that extracellular α-syn within the CSF and dura can directly engage border immune and lymphatic compartments, positioning the dura mater as an active site of disease-relevant protein handling and immune regulation rather than a passive drainage interface.

Our findings suggest that border-associated macrophages may represent a key cellular link between extracellular α-syn accumulation and impaired CNS clearance. In both our acute and chronic models, α-syn exposure was associated with dural macrophage activation, while chronic CSF-derived α-syn selectively elevated TNF without broad induction of other inflammatory cytokines such as IL-1β or IFNγ. This aligns with meta-analyses reporting elevated TNF in the CSF and peripheral blood of patients with PD, supporting TNF as a disease-relevant inflammatory mediator across central and peripheral compartments (Qu et al., 2023). This is also consistent with previous work showing that α-syn can induce inflammatory activation states in border-associated macrophages and activate macrophages in CSF-draining LN (Liu et al., 2023; Schonhoff et al., 2023; Xu et al., 2025). Together with our finding that TNF directly impairs LEC structure and function, these data suggest that macrophage-associated TNF is not merely a marker of inflammation, but a plausible mediator linking α-syn handling at CNS borders to lymphatic dysfunction (Kouchaki et al., 2018; Sriram et al., 2002). Although our data support border-associated macrophages as a likely source of TNF following α-syn exposure, we cannot exclude contributions from other dural immune populations, including T cells, mast cells, dendritic cells, or other myeloid populations (Lindestam Arlehamn et al., 2020; Mamuladze et al., 2025; Nakae et al., 2005; Rustenhoven et al., 2021; Serbina et al., 2003).

There are also several important considerations. While our human data strongly support dural α-syn pathology as a disease-relevant feature of PD, larger postmortem cohorts will be required to determine its prevalence, temporal relationship to parenchymal pathology, and specificity across α-synucleinopathies. Future studies should also examine whether similar meningeal α-syn pathology occurs in Lewy body disease, multiple system atrophy, and cases with mixed neurodegenerative pathologies. Secondly, the acute PFF and chronic AAV-SP2-α-syn models used here were designed to isolate the effects of extracellular α-syn at CNS borders and do not fully reproduce the progressive neuronal origin and spread of α-syn pathology in PD. Importantly, these clearance and immune alterations occurred in the absence of overt brain parenchymal α-syn accumulation, gliosis, dopaminergic neuron loss, or behavioral impairment, suggesting that CNS border dysfunction can occur without detectable brain pathology. This raises the possibility that impaired lymphatic clearance and α-syn accumulation at CNS borders may form a feed-forward cycle, whereby extracellular α-syn disrupts drainage pathways and thereby further limits its own clearance. Thirdly, although our tracer drainage assays measure cumulative CSF efflux to cervical LNs and therefore capture drainage through multiple routes, our tissue analyses focused on dorsal and lateral dural regions associated with the dural sinuses. Basal meningeal lymphatics and nasopharyngeal lymphatic routes also contribute to CNS waste clearance and may therefore be similarly affected by CSF-derived α-syn (Chayama et al., 2026; Jin et al., 2025; Yoon et al., 2024). Future studies should determine whether α-syn–driven immune activation and lymphatic junctional remodelling also occur across these additional drainage pathways. However, the convergence of human dura pathology, mouse functional drainage deficits, macrophage-associated TNF production, and direct lymphatic endothelial disruption supports a model in which α-syn accumulation at CNS borders contributes to clearance failure in PD.

These findings reveal CNS border tissues as actionable therapeutic sites in PD. Enhancing meningeal lymphatic drainage, improving CSF α-syn clearance, modulating border-associated macrophage activation, or limiting TNF-mediated lymphatic dysfunction may represent strategies to restore CNS waste clearance and interrupt the feed-forward cycle of α-syn accumulation, inflammation, and drainage failure (Alfaidi et al., 2025; Rustenhoven et al., 2023; Tong et al., 2023). Importantly, our data suggest that α-syn–driven lymphatic dysfunction is likely multifactorial, involving both direct effects of α-syn on LECs and indirect effects mediated through macrophage-associated TNF. Therefore, targeting TNF alone could attenuate inflammatory lymphatic dysfunction but may not fully restore clearance if α-syn aggregates continue to directly impair lymphatic endothelial integrity. More broadly, our work shifts CNS borders from passive routes of waste removal to active sites of PD pathogenesis, identifying the dura mater and its immune–lymphatic networks as accessible targets for modifying disease-associated clearance failure.

## Materials and methods

### Human brain tissue collection and processing

The postmortem human dura tissue used in this study was obtained from the Neurological Foundation of New Zealand Human Brain Bank in the Centre for Brain Research at the University of Auckland. The human tissue was donated with consent, and its use was approved by the University of Auckland Human Participants Ethics Committee (14/NTA/208). All cases were examined by a neuropathologist and were classified based on neurological abnormalities, or lack thereof, in neurologically normal cases (Table S1).

### Paraffin-embedded immunohistochemistry

Standard paraffin immunohistochemistry protocols were carried out on paraffin-embedded sections as described previously (Singh-Bains et al., 2021; Stevenson et al., 2020). Slides were heated to 60°C for 1 h on a heating plate to melt the paraffin wax. Slides were cleared in xylene (two times for 30 min) and rehydrated in an ethanol series: two times with 100% (15 min), one time with 95% (10 min), one time with 80% (10 min), one time with 75% (10 min), followed by three times for 5 min in distilled H_2_O. Heat-induced antigen retrieval was performed with a Tris-EDTA (pH 9.0) buffer in a pressure cooker (2100 Antigen Retriever, Aptum Biologics Ltd.) for 20 min at 121°C and left to cool for 1.5 h. Once cooled, slides were washed three times for 5 min in PBS and permeabilized in 0.1% Triton X-100 in PBS (PBS-T) for 15 min at 4°C. The sections were then blocked for nonspecific secondary antibody binding for 1 h in 10% serum from the species in which the secondary antibody was raised. Sections were subsequently incubated with primary antibodies diluted in 1% serum and left overnight in a humidified chamber at 4°C. Subsequently, slides were washed three times for 5 min in PBS. Secondary antibodies were diluted in 1% serum and incubated on the sections for 3 h at room temperature. Following this, sections were washed three times for 5 min in PBS. Sections were incubated for 5 min in PBS containing a 1:20,000 dilution of Hoechst 33342 to counterstain nuclei and subsequently washed three times for 5 min in PBS. Sections were coverslipped with Prolong Gold (#P36930; Molecular Probes). Sections were sealed around the edges of the coverslip using nail polish and stored at 4°C in the dark until imaged. A list of antibodies can be found in Table S2.

### Animals

Male or female WT mice (C57BL/6J background) were bred in-house. Mice were housed in a temperature and humidity-controlled environment with a 12-h light/dark cycle. They were provided with food and water ad libitum. Mice were used for experiments at 2–3 mo of age. All mice were WT, and thus no backcrossing was performed. All mice were habituated for at least 72 h in the animal facility before the start of experimentation. Sample sizes were chosen on the basis of a power analysis using estimates from previously published experiments (Rustenhoven et al., 2021; Rustenhoven et al., 2023). All experiments were approved by the Animal Ethics Committee at the University of Auckland.

### AAV vectors

AAV plasmid expressing GFP or human WT α-syn under the control of the chicken beta actin/CMV hybrid promoter and containing a woodchuck posttranscriptional regulatory element (WPRE), bovine growth hormone polyadenylation signal, and scaffold attachment region flanked by AAV2 inverted terminal repeats was packaged into AAV9 capsids (Lawlor et al., 2009). Vector titers were determined using primers to WPRE as described previously (Lawlor et al., 2009). AAV9-CAG-GFP and AAV9-CAG-Sp2- α-syn were supplied by Vector Labs based on a previous construct (Prymaczok et al., 2024). This allowed for constitutively secreted α-syn by fusing a removable short signal peptide to the N-terminal end of human WT α-syn (Prymaczok et al., 2024).

### ICM injections

Mice were anesthetized via intraperitoneal ketamine (75 mg/kg) and medetomidine (1 mg/kg). The fur from the head and neck regions was removed with depilatory cream, and the underlying skin was disinfected with 2% chlorhexidine and 70% ethanol. To prevent drying, an ophthalmic solution was applied to the eyes, and the head was securely fixed in a stereotaxic frame. Mice then received a subcutaneous injection of 2 mg/kg bupivacaine for postoperative analgesia overlying the intended incision site. A small incision in the neck was made, and the muscle layers were retracted with fine surgical hooks exposing the CSF-filled cisterna magna. Using a Hamilton syringe coupled to a 33-gauge needle, injections of 3 μl AAV9-CAG-GFP control, 3 μl of AAV9-SP2-α-syn (both at 10^13^ genome copies per ml in sterile PBS), 2.5 μl of OVA conjugated to Alexa Fluor 594 (OVA-A594; 1 mg/ml in sterile PBS), 3 μl a fluorescent cocktail mixture of cadaverine conjugated to Alexa Fluor 488 (cadaverine-488; 5 mg/ml), Texas-red fluorescent dextran (3 kDa) (dextran-594; 5 mg/ml), and OVA conjugated to Alexa Fluor 647 (OVA-647; 1 mg/ml), 2 μl of lightning linked-594 α-syn PFFs (100 μM), or 2 μl of α-syn PFFs (100 μM) were administered into the CSF via an ICM injection at a controlled rate of 1 μl/min, with an additional 2-min retention to prevent backflow. The syringe was carefully retracted, and the incision site was closed with Leukosan Adhesive Skin Glue. Following ICM injections, mice were subcutaneously administered 2.5 mg/kg Atipamezole hydrochloride for anesthesia reversal and allowed to recover on a heat pad until fully awake.

### Immunohistochemistry

Mice were euthanized using a lethal dose of 150 mg/kg pentobarbitone via intraperitoneal injection and then transcardially perfused with ice-cold heparin (10 U/ml) in PBS for exsanguination. For LN collections, mice were positioned supine, and dCLNs were collected via gentle retraction of the submandibular glands and collection laterally on either side of the trachea with fine surgical forceps. Subsequently, mice were decapitated just posterior to the occipital bone, and the overlying skin and muscle were removed from the skull. Using fine surgical scissors, the skull cap containing the skull bone and the attached dura mater was then carefully removed by clockwise incisions, beginning and ending at the occipital bone. The brain was then removed from the cavity. For ACE point analysis, mice were first perfused with ice-cold heparin (10 U/ml) in PBS, then 4% PFA, and the skull caps, containing the dura, were collected as described but with gentle downward pressure on the skull when collecting to ensure good bridging vein collection. LNs were then drop-fixed in 4% PFA for 24 h at 4°C and washed in PBS. The fixed nodes were placed in 30% sucrose for another 24 h at 4°C before embedding in optimal cutting temperature (OCT) and rapid freezing over dry ice. Frozen LNs were cut (30-µm thick sections) in a cryostat and collected onto Superfrost slides. Roughly 10–20 sections were cut for each node, spanning the entirety of the node. Skull caps containing meningeal whole mounts were drop-fixed in 4% PFA for 2 h, and the dura mater was gently peeled from the inner surface of the skull using fine surgical forceps and then placed in PBS until further use. Brains were drop-fixed in 4% PFA for 48 h at 4°C, then placed in 30% sucrose in PBS until the brain had completely sunk, usually within 24–48 h, at 4°C. The brains were then embedded in OCT reagent, rapidly frozen on dry ice, and stored at −20°C. Coronal cryosections (40–100-µm thick sections) were cut using a cryostat, and the resulting free-floating sections were preserved in PBS until ready for use. Free-floating brain sections and meningeal whole mounts were subjected to blocking and permeabilization for 1 h at room temperature in 24-well plates, with continuous agitation, using donkey immunobuffer (PBS with 1% Donkey serum and 0.2% Triton X-100) at room temperature. Slides containing LNs were marked with the outline of a hydrophobic pen and blocked/permeabilized for 30 min with donkey immunobuffer at room temperature. Brains and meninges were then incubated with primary antibodies overnight at 4°C with gentle agitation in 24-well plates, and LNs on slides in a humidified chamber. The list of antibodies used can be found in Table S2. The following day, all tissues were washed three times with PBS-T. Sections were then incubated with secondary antibodies and DAPI (1 µg/ml) at room temperature for 2 h with agitation for brains and meninges or on slides for LNs. Finally, sections were washed with PBS-T, and brains and meninges were mounted on Superfrost Plus slides (Thermo Fisher Scientific) and coverslipped using Anti-Fade Fluorescence Mounting Medium.

### Imaging and analysis

Widefield images were acquired using a Slideview VS200 slide scanner equipped with a 4× objective lens for macroscopic overview images, and the 20× (0.5 NA UPLFLN) objective lens was used for high-resolution images. For tissue sections larger than the microscope objective’s field of view, adjacent fields were acquired with the SLIDEVIEW VS200 slide scanner and automatically stitched using the VS200 acquisition software. Confocal imaging was performed using the Leica LSM800 and 40× objective or the Olympus FV4000 and 20×, 40×, or 60× objective. Image analysis was performed using the FIJI plugin for ImageJ (National Institutes of Health). Analysis of glymphatic influx was performed as described previously (Rustenhoven et al., 2023) using thresholded cadaverine-488, dextran-594, and OVA-647 coverage in brain sections as a percentage of overall brain-slice area. For each mouse, 8–12 brain sections (100 µm each) were analyzed and averaged to generate the value for a single mouse. Analysis of meningeal Lyve1, GFP, or α-syn coverage was performed as described previously (Rustenhoven et al., 2023) using thresholded coverage as a percentage of the overall meningeal area. α-syn load in postmortem tissue was determined using thresholded coverage as a percentage of total area of tissue. Zipper-like junctions were identified as continuous junctional staining along cell–cell borders, typically associated with elongated cell morphology. Button-like junctions were characterized by discontinuous junctional staining that was not aligned parallel to the cell borders, together with an oak leaf–like cell shape. Junctional patterns that did not clearly fit either of these categories were classified as mixed as previously described (Hong et al., 2020). Analysis of lymphatic drainage and Lyve1 coverage in LNs was performed as described previously (Rustenhoven et al., 2023) using thresholded OVA-594, cadaverine-488, dextran-594, and OVA-647, or Lyve1 coverage as a percentage of overall LN area. For each mouse, 10–20 sections were analyzed from each LN and averaged to generate the value for a single mouse.

### Multiplex cytokine analysis

Cytokine levels in CSF taken from postmortem mice treated with 3 μl AAV9-GFP control, 3 μl of AAV9-SP2-α-syn (both at 10^13^ genome copies per ml in sterile PBS) after 2 mo were measured using the Bio-Plex Pro Mouse Cytokine 23-plex Assay (Cat# M60009RDPD; Bio-Rad) according to the manufacturer’s instructions. Briefly, samples and standards were incubated with magnetic capture beads specific to 23 mouse cytokines in a 96-well filter plate for 30 min at room temperature with shaking. After washing, biotinylated detection antibodies were added and incubated for 30 min with shaking, followed by the addition of streptavidin-phycoerythrin and a final 10-min incubation. Plates were washed between each step using a magnetic plate washer. Data acquisition was performed using a MAGPIX Multiplexing system. Cytokine concentrations were determined from a five-parameter logistic standard curve generated using known standards, and values were expressed as pg/ml.

### α-Syn PFFs preparation

α-Syn PFFs were created in-house as previously described (Patterson et al., 2019). Briefly, α-syn monomers (RP-010; Proteos) were thawed on ice and centrifuged at 15,000 × *g* for 10 min at 4°C. Protein concentration was measured using the absorbance at 280 nm with a microvolume spectrophotometer (NanoDrop 1000; Thermo Fisher Scientific). α-Syn monomers were subsequently diluted in 1× PBS to a final concentration of 5 mg/ml. The monomers were then placed in an orbital thermomixer (Eppendorf) at 37°C for 7 days, shaking at 1,000 RPM to create fibrils. Following the 7 days, appropriate amounts were set aside for characterization and quality assurance. The remaining fibrils were stored at −80°C.

### Electron microscopy

For visualization of α-syn fibrils with transmission electron microscopy, α-syn preparations were diluted in PBS to a concentration of 1 μM. Formvar/carbon-coated copper 300 mesh grids (Sigma-Aldrich) were placed on top of 10-μl droplets of the diluted α-syn preparations and subjected to adsorption for 5 min. Following this, the preparations were negatively stained with 2% aqueous uranyl acetate for 2 min α-syn fibrils were imaged using a Tecnai G1 Spirit Twin transmission electron microscope mounted with an Olympus-Soft Imaging Systems Morada digital camera.

### Endotoxin quantification

The quantification of endotoxins in the PFF monomers and PFF aggregates was carried out using the Pierce Chromogenic Endotoxin Quant Kit (A39552; Thermo Fisher Scientific) as per the manufacturer’s instructions. Briefly, the endotoxin standards, samples, and blanks were all run in triplicate. To run the standards and samples, a 96-well plate was pre-equilibrated by being slightly submerged in a 37°C water bath and remained there for the remainder of the assay. Endotoxin standard, blanks, and α-syn preparations (100 nM) were added to each well, followed by the addition of reconstituted Amebocyte Lysate Reagent, and incubated for 60 min. Next, Reconstituted Chromogenic Substrate Solution was added to each well and incubated for 6 min. After 6 minutes, Stop Solution (25% acetic acid) was added. Optical density (OD) at 405 nm was read immediately after assay completion using a Synergy 2 Multi-Mode Microplate Reader (BioTek). Concentrations of endotoxins were calculated using a standard curve with a coefficient of determination, R2, ≥0.98.

### Thioflavin T assay

In a clean microcentrifuge tube, 5 μl of PFFs were added to 245 μl of 1× PBS to dilute the fibrils 1:50. Next, 250 μl of 1× PBS was added to a separate microcentrifuge tube to act as a negative control. For the monomer control, 5 μl of monomer was added to 245 μl of 1× PBS. Next, 250 μl of 25 μM thioflavin T (Sigma-Aldrich) was added to each sample and gently mixed. Two replicates, 200 μl each, were placed into a 96-well plate (Nunc) and kept in the dark to prevent photobleaching. The α-syn was incubated for 1 h at room temperature with thioflavin T, and the plate was read at an excitation of 450 nm and emission of 510 nm.

### Seed amplification assay (SAA)

We employed an in-house SAA protocol optimized for fresh-frozen postmortem human brain tissues, adapted from previously described methods (Schmitz et al., 2016; Wiseman et al., 2025). This assay uses RT-QuIC to amplify and detect α-syn aggregates.

### Buffer preparation for SAA

All buffers were filter-sterilized (0.22 µm) and used within 3 mo. The homogenization buffer (0.1% SDS in PBS, pH 7.4) was vortex-mixed and stored at 4°C. Base mix preparation was scaled for 500 reactions to minimize pipetting error, combining ddH_2_O, 5×PBS (pH 6.9), NaCl, and EDTA.

### Tissue homogenization and CSF preparation

Fresh-frozen dura tissue was weighed and diluted to 0.625% (wt/vol) in homogenization buffer, then homogenized using a Bullet Blender (two 4-min rounds, level 8). After homogenization, samples were centrifuged (10,000 × *g*, 10 min), and both soluble (supernatant) and insoluble (pellet) fractions were aliquoted and stored at −80°C. CSF was collected from the choroid plexus and lateral ventricles, diluted 1:2 in homogenization buffer, and stored at −80°C.

### RT-QuIC assay

RT-QuIC assay was based on previous studies (Wiseman et al., 2025). Briefly, α-syn monomers (5 mg/ml) were filtered through 100-kDa filters to remove aggregates and diluted to 0.37 mg/ml in PBS. Reactions were assembled in 96-well optical plates with 85 μl reaction buffer and 15 μl of sample, loaded in triplicate, avoiding air bubbles. Plates were sealed and run on a CLARIOstar plate reader at 42°C for 60 h, using 1-min double-orbital shaking cycles followed by stationary incubation. Fluorescence was measured every 10 min. Optical settings were set at excitation 445 ± 5 nm and emission 480 ± 5 nm, with a dichroic at 462.5 nm.

### Single-cell isolation and flow cytometry

Unfixed dural meninges were removed from the skull cap using fine forceps and kept on ice-cold DMEM throughout the collection process. The meninges were digested for 15 min at 37°C with constant agitation using 1 ml of pre-warmed digestion buffer (DMEM, 2% FBS, 1 mg/ml collagenase VIII, and 0.5 mg/ml DNase I). At completion, samples were triturated five times with a 1-ml pipette tip and filtered through a 70-µm cell strainer. Collagenase was then neutralized by the addition of 1 ml of DMEM containing 10% FBS. Following centrifugation at 300 × *g* for 5 min, the samples were resuspended in ice-cold fluorescence-activated cell sorting (FACS) buffer (PBS with 1 mM EDTA and 1% BSA) and kept on ice until use. For surface staining, single-cell suspensions were incubated in FACS buffer containing anti-CD16/32 (FC block), diluted 1:50 for 5 min, followed by the addition of fluorescently conjugated antibodies for 20 min at 4°C. Subsequently, the samples were washed in FACS buffer and run on a Cytek Aurora 5 laser system (School of Biological Sciences, University of Auckland) and analyzed using FlowJo software (Tree Star). The list of antibodies used for flow cytometry can be found in Table S2.

### Mouse dural fibroblast culture

Adult female/male WT mice (2–3 mo old) were euthanized humanely by CO_2_ inhalation. Mice were decapitated just posterior to the occipital bone, and the overlying skin and muscle were removed from the skull. Using fine surgical scissors, the skull cap was then carefully removed by clockwise incisions, beginning and ending at the occipital bone. The dura mater of the meninges was isolated by carefully peeling away from the skull cap using fine forceps and washed in fibroblast media. All procedures were performed in a cell culture biosafety cabinet. The meninges then underwent a 15-min digestion at 37°C using 1 ml of pre-warmed digestion buffer (DMEM/F12 with 2% FBS, 1 mg/ml collagenase VIII from Sigma-Aldrich, and 0.5 mg/ml DNase I from Sigma-Aldrich). The digested material was triturated five times with a 1 ml pipette, then filtered through a 70-μm cell strainer, and the collagenase was neutralized with 1 ml of fibroblast media. The resulting cell suspension was centrifuged at 300 × *g* for 5 min, and the cell pellet was resuspended and plated into a 6-well plate, with one dura per well, in fibroblast media. The culture medium was changed every 3–4 days, and after 2–2.5 wk, confluent cultures were observed. For passage or plating, cells were washed with PBS, dissociated using 0.25% Trypsin-EDTA for 5–10 min at 37°C. The trypsin was neutralized with twice the volume of fibroblast media and centrifuged at 300 × *g* for 5 min. The cell pellet was then resuspended in fibroblast media, and cells were counted using a hemocytometer and Trypan Blue exclusion. The fibroblasts were seeded at a density of 10,000 cells per well in 96-well plates.

### ELISA

Mouse dural fibroblasts were treated with AAV9-CBA-GFP (1 × 10^11^ GC/ml), AAV9-CBA-WT α-syn (1 × 10^11^ GC/ml), AAV9-CAG-GFP control, or AAV9-CAG-SP2-α-syn (both at 1 × 10^11^ GC/ml) for 7 or 14 days. Conditioned media was collected and human α-syn secretion levels were measured using the Human α-Synuclein ELISA Kit (Cat# KHB0061; Thermo Fisher Scientific) according to the manufacturer’s instructions.

### Cytometric bead array

Conditioned media samples from THP-1 monocytes and macrophages treated with vehicle (PBS) or α-syn (500 nM PFFs) were spun at 200 × *g* for 5 min, and the supernatant was collected and stored at −20°C. The concentration of TNF (Enhanced Sensitivity Flex Set, 561516; BD Biosciences) was measured using a cytometric bead array (BD Biosciences) as per the manufacturer’s instructions. Cytometric bead array samples were run on an Accuri C6 flow cytometer (BD Biosciences). Data were analyzed using FCAP-array software (version 3.1; BD Biosciences) to convert fluorescent intensity values to concentrations using an eight-point standard curve (0–200,000 fg/ml).

### Human iPSC lines

The human iPSC lines (Table S3) were purchased from the American Type Culture Collection (ATCC) or The Jackson Laboratory. The use of iPSCs in this project was covered under Hazardous Substances and New Organisms approval number GMD102459.

### iPSC maintenance

iPSCs were maintained at 37°C and 5% CO_2_ in StemFlex medium in Geltrex-coated plates (12.5 µg/cm^2^). The StemFlex medium was refreshed every 2–3 days. iPSC’s were passaged when ∼60–80% confluent. To passage, iPSCs were washed in PBS and brought to suspension with Versene. The iPSCs were gently triturated three to five times in the Versene solution until the cell suspension reached small clumps of ∼5–15 cells, and iPSCs were split at a ratio of 1:6–1:40 depending on the line. StemFlex medium was refreshed the following day to remove Versene from the medium.

### LEC differentiation protocol

LECs were differentiated as described previously (Gröger et al., 2004; Hitpass Romero et al., 2025; Nicenboim et al., 2015). When 60–80% confluent (day 0), iPSCs were brought into a single-cell suspension with Accutase. Accutase was inactivated with DMEM/F12, counted, and centrifuged at 200 × *g* for 4 min. iPSCs were resuspended in StemFlex medium + 10 µM ROCK inhibitor (Y-27632) and were seeded at 37,000 cells/cm^2^ into a Geltrex-coated 12- or 6-well plate and incubated at 37°C and 5% CO_2_ for 24 h. On day 1, the StemFlex medium + Y-27632 was replaced with mesoderm induction media (Table S4) for three days. On day 4, the cultures were placed in lymphatic induction media (LIM) (Table S4). LIM was refreshed on day 5, generating a heterogenous cell culture on day 6; thus, a magnetic-activated cell sorting (MACS)-based enrichment step was performed. On day 6, cells were brought into a single-cell suspension with Accutase, which was subsequently inactivated with DMEM/F12 + 10% FBS. The inactivated cell suspension was passed through a 70-µm cell strainer and washed through with an additional 2 ml of DMEM/F12. Cells were centrifuged at 200 × *g* for 4 min, and the MACS enrichment was carried out using a MACS MultiStand, MidiMACS separator, and a CD31 Microbead kit according to the manufacturer’s instructions. Briefly, the supernatant was completely aspirated, and the cell pellet was resuspended in 60 μl of DMEM/F12 followed by 20 μl of FcR Blocking reagent and 20 μl of CD31 Microbeads. The cell suspension was gently resuspended and was incubated at 4°C for 15 min. Following the incubation, 1 ml of DMEM/F12 was added, and the cell suspension was centrifuged at 200 × *g* for 4 min. During this process, an LS column was prepared by washing through 3 ml of DMEM/F12 in the magnetic field of a MidiMACS separator. The cell pellet was resuspended in 1 ml DMEM/F12 and was added to the prepared column. Once the solution had fully flowed through, the column was washed three times, each with 3 ml DMEM/F12. After the last wash, the LS column was removed from the separator, placed on top of a collection tube, and 5 ml of DMEM/F12 was added and immediately flushed through with a plunger. The CD31^+^ cell suspension was centrifuged at 200 × *g* for 4 min and resuspended in VEGF-C (100 ng/ml) supplemented endothelial media (sEM; ScienCell; Table S4). The CD31^+^ cells were plated in 0.1% gelatin-coated plates at 150,000 cells/cm^2^.

### iLEC passaging and maintenance

iLECs were maintained at 37°C and 5% CO_2_ in sEM, which was refreshed every 3–4 days. iLECs were passaged when ∼60–90% confluent. To passage, cells were brought into single-cell suspension with trypsin. Trypsin was neutralized with DMEM/F12 + 10% FBS, and cells were centrifuged at 300 × *g* for 5 min. Viable cells were resuspended in sEM and plated into gelatin-coated plates at a ratio of 1:2–1:3 for further expansion, or seeded at specific cell densities for each experiment. Where not otherwise mentioned, iLECs were seeded at 10,000 cells/well in 96-well plates and 15,000 cells/well in 48-well plates. All iLECs used in experiments were between passages 2–5, as cell senescence was observed thereafter.

### iLEC treatments with TNF and α-syn

iLECs were seeded into 96-well plates at a density of 15,000 cells per well and treated with vehicle (0.1% BSA), α-syn PFFs (500 nM), or TNF (10 ng/ml; PeproTech) for 24, 48, or 72 h. At the designated time points, cells were collected for flow cytometry or fixed with 4% PFA for immunocytochemistry. For flow cytometry, the same vehicle was used for both TNF and α-syn PFFs treatment.

### Microfluidic sprouting assays

identX microfluidic chips (Aim Biotech) and 10-μl pipette tips were pre-cooled at 4°C to minimize premature gelling. Fibrin/thrombin gels were prepared by the addition of 25 mg/ml fibrinogen to 10 U/ml thrombin on ice. The central gel chamber of identX microfluidic chips was loaded with 10 μl using cooled chips and pipette tips. Chips were incubated at 37°C for 30 min to enable gelling. The media chamber for iLEC seeding was pre-coated with 15 μl of 0.1% gelatin-based coating solution for 5 min at room temperature, and the other media channel was hydrated with 15 μl sEM. The gelatin-based coating solution was washed and removed by the addition of 70 μl to one media port and 50 μl to the opposite attached media port. This was repeated three times, each time removing the excess media. Media ports were then hydrated with 70 μl of sEM in one port and 50 μl of sEM in the opposite attached port. iLECs were seeded in the cell gelatin-coated chamber at 60,000 cells/well in 20 μl sEM and allowed to adhere overnight. The next day, sEM was removed from all ports, and the iLEC-containing media ports received non-supplemented EM (50 μl in one port, 30 μl in the other port) containing only the base formulation without FBS or ECGS, while the opposite media ports received complete EM further supplemented with 100 ng/ml VEGFC, Ang1, and HGF (70 μl in one port, 50 μl in the other port). For treatment conditions, iLECs were exposed daily to either α-syn PFFs (500 nM) or TNF (10 ng/ml; PeproTech), which were added directly to non-supplemented EM. This approach generates a concentration gradient of lymphangiogenic factors to promote sprouting, and a small pressure difference to generate interstitial flow. This approach was repeated daily for 7 days. Chips were stained with Actin Green (2 drops/ml) and DAPI (1 µg/ml) for 2 h at room temperature, then washed with PBS-T, all via addition of 50 μl to one port, 30 μl to the other port on one side, and 70 μl to one port, 50 μl to the other port on the other side to visualize lymphatic structures.

### THP-1 monocytes and macrophages

Human monocytic THP-1 cells (ATCC TIB-202) were cultured in RPMI-1640 medium supplemented with 10% FBS, 2.5g/l D-glucose, and 0.05 mM 2-mercaptoethanol at 37°C in a humidified atmosphere containing 5% CO_2_. Cells were maintained at a density between 2 × 10^5^ and 1 × 10^6^ cells/ml and passaged every 2–3 days to ensure viability and consistency. For differentiation into macrophage-like cells, THP-1 monocytes were seeded into 96-well plates at a density of 50,000 cells per well and left as a monocyte culture, or differentiated into macrophages with 100 nM PMA for 24 h. Following PMA exposure, cells were washed once with RPMI-1640 to remove residual PMA and cultured in fresh PMA-free RPMI-1640 medium for an additional 24 h to allow for recovery and maturation. Macrophage differentiation was confirmed by visual assessment of cell adherence and morphology under a phase-contrast microscope. Monocytes and macrophages in 96-well plates were treated with 500 nM α-syn PFFs or vehicle (PBS) for either 4 or 24 h. At the designated time points, conditioned media were collected for downstream analysis, and cells were fixed with 4% PFA for immunocytochemistry.

### LDH cytotoxicity assay

Cytotoxicity was assessed using the Cytotoxicity Detection Kit (11644793001; Roche) according to the manufacturer’s instructions. Briefly, THP-1 cells were seeded into 96-well plates and treated under the indicated experimental conditions. At the end of the treatment period, culture supernatants were collected and transferred to an optically clear 96-well plate. Freshly prepared LDH reaction mixture was added to each well at a 1:1 ratio and incubated at room temperature protected from light. Absorbance was then measured using a microplate reader at 490/492 nm, with a reference wavelength above 600 nm. Background, low-control/spontaneous LDH release, and high-control/maximum LDH release wells were included for each experiment. Cytotoxicity was calculated from background-corrected absorbance values relative to the low and high controls.

### Immunocytochemistry

Cell cultures were fixed with 4% PFA at room temperature for 15 min. This was followed by three washes with PBS-T to permeabilize the cells for subsequent staining. Primary antibodies were diluted accordingly in PBS containing 0.2% Triton X-100, 1% donkey serum, and 0.05% Proclin (collectively referred to as donkey immunobuffer). Cells were incubated overnight at 4°C with gentle agitation. The next day, cells were washed three times with PBS-T for a total of 15 min before addition of secondary antibodies. Secondary antibodies and nuclear stain DAPI (1 µg/ml) were diluted in donkey immunobuffer and were added for 2 h at room temperature with gentle agitation. Antibodies used can be found in Table S2.

### Image capture and analysis

The ImageXpress Micro XLS high content screening system was used to capture fluorescent images of cultured cells in 96-well plates. The 20× (0.45 NA) objective lens was used. The exposure times and focus were optimized for each plate to account for antibody efficiency. The quantitative analysis of image measurements was conducted with the FIJI package for ImageJ. For the coverage measurements, VE-Cadherin– and α-syn–positive signals were identified by thresholding of images and quantified as area of signal/total area of site, resulting in a percentage area measurement. The same parameters were applied across all groups during imaging and threshold settings to mitigate experimental bias. The average percentage area for each condition was normalized relative to the average cell count, determined by the number of DAPI-positive cells. Given the varied baseline expression of each parameter across individual cases, data were normalized based on the vehicle control within each respective case. Cell scoring analysis was carried out using the MetaXpress in-built cell scoring software (version 6.5.5). Briefly, images from the DAPI channel served as a reference, and scoring was conducted in comparison with the corresponding FITC image. The GFP signal’s minimum and maximum width were adjusted to exclude any autofluorescent debris while ensuring cells were counted individually. Additionally, the intensity above background was optimized to minimize background noise. The same parameters were applied across all groups within each case to mitigate experimental bias.

### Behavioral testing

Behavioral testing was performed on C57BL/6J mice following ICM of AAV9-GFP or AAV9-α-syn. Mice were randomly assigned to experimental groups and, where possible, groups were balanced for sex and litter. Behavioral testing was conducted in two cohorts, each containing both GFP- and α-syn–injected animals. The same experimenter performed all handling and behavioral testing throughout the study, and analyses were performed blinded to treatment group where possible.

The behavioral battery was performed in the order of increasing expected stress: open field test, novel object recognition, and rotarod. Testing was conducted at a consistent time of day in the early morning, with start times maintained within 60 min across testing days. Animals were habituated to the experimenter before behavioral testing and were transported to the testing room at least 60 min before each session to acclimatize. Behavioral blocks were separated by 1–3 days where required. All arenas, objects, and equipment were cleaned with 70% ethanol between animals, with feces and urine removed and equipment allowed to dry for 3–5 min to minimize residual olfactory cues. Behavior was recorded using an overhead camera and analyzed using EthoVision XT 17 unless otherwise stated.

### Open field test

Locomotor activity and anxiety-like behavior were assessed using the open field test. Mice were placed individually into the center of a square open field arena with black walls (40 × 40 cm) and allowed to freely explore for 5 min. At the end of the trial, mice were returned to their home cage and the arena was cleaned before the next animal was tested. Total distance travelled, time spent in the center and peripheral zones, and center zone entries were quantified from video recordings using EthoVision XT 17.

### Novel object recognition

Recognition memory was assessed using the novel object recognition test in the same square arena used for open field testing. The task was performed over 3 days. On the first 2 days, mice were habituated to the empty arena for 5 min per day. On the third day, mice underwent a 5-min training session in which two identical familiar objects were fixed to the arena floor. Mice were placed into the center of the arena facing away from the objects and allowed to freely explore. Following training, mice were returned to their home cage for a retention interval of at least 1 h. During the test session, one familiar object was replaced with a novel object of different shape and color, and mice were again allowed to explore the arena for 5 min. Object exploration was quantified from overhead video recordings using EthoVision XT 17. Recognition memory was expressed as the discrimination index.

### Rotarod

Sensorimotor performance and motor learning were assessed using an accelerating rotarod paradigm. Testing was performed using an IITC rotarod fitted with a 3 cm diameter rod suitable for mice. Mice were placed on the stationary rod facing away from the experimenter and allowed to grip the rod with all four paws before each trial began. Animals were trained over three consecutive days, with three trials per day. During each trial, the rod accelerated from 4 to 40 rpm over 5 min. A minimum inter-trial interval of 10 min was maintained to reduce fatigue.

Following training, mice underwent a test session using the same accelerating protocol. Each mouse received three trials, separated by at least 10 min. The primary outcome measure was latency to fall, defined as the time from the start of the trial until the animal fell from the rod, with a maximum trial duration of 300 s. If an animal gripped the rod and passively rotated without ambulating, latency was recorded after no more than two complete passive rotations. Latency to fall was averaged across the three test trials for each animal.

### Reanalysis of published scRNA-seq datasets

#### Border-associated myeloid scRNA-seq

To examine *Tnf* induction in border-associated macrophages following α-syn exposure, we reanalyzed a publicly available scRNA-seq dataset of midbrain myeloid cells from a substantia nigra α-syn overexpression model (Schonhoff et al., 2023); GEO accession GSE178498). This dataset comprises four samples generated 4 wk after stereotaxic injection of AAV2-GFP (two replicates) or AAV2-α-syn (two replicates) into the substantia nigra pars compacta. Briefly, in the original study, mononuclear cells were isolated from three to four pooled ventral midbrains per sample and sorted as CD45^+^CD11b^+^CX3CR1^+^Ly6C^−^Ly6G^−^NK1.1^−^ cells, profiled on the 10X Genomics Chromium platform (Single Cell 3′ Reagent Kit version 2), and sequenced to an average depth of 20,000 reads per cell on an Illumina NovaSeq; reads were mapped to the mm10 transcriptome and count matrices generated with Cell Ranger (version 3.1.0). For the present reanalysis, the Cell Ranger–processed filtered feature-barcode matrices for each sample were downloaded from GEO and imported into R, and a Seurat object was created for each sample with CreateSeuratObject, retaining genes detected in at least 3 cells (min.cells = 3) and cells with at least 200 detected genes (min.features = 200). Condition (GFP or α-syn) labels were added as metadata, and the four objects were combined into a single object with merge. The percentage of mitochondrial reads was calculated per cell (PercentageFeatureSet, pattern = “^mt-”), and low-quality cells were removed by retaining only cells with more than 200 and fewer than 6,000 detected genes (nFeature_RNA) and <10% mitochondrial content. Counts were log-normalized (NormalizeData), the 2,000 most variable genes were identified by variance-stabilizing transformation (FindVariableFeatures), and expression values were scaled across genes (ScaleData) prior to principal component analysis (RunPCA). The first 10 principal components were used for uniform manifold approximation and projection (RunUMAP, dims = 1:10), shared-nearest-neighbor graph construction (FindNeighbors, dims = 1:10), and Louvain clustering (FindClusters, resolution = 0.3). Clusters were annotated by canonical myeloid markers (*Cx3cr1*, *Sall1*, *Clec12a*, and *Mrc1*), which identified cluster 5 as meningeal/perivascular macrophages (*Clec12a*^*+*^, *Mrc1*^*+*^, and *Sall1*^*−*^). This cluster was subsetted (subset), and Tnf expression was compared between AAV2-GFP and AAV2-α-syn cells.

#### Dural endothelium scRNA-seq

To assess the capacity of LECs to respond to TNF, we reanalyzed a published dural stromal scRNA-seq dataset (Rustenhoven et al., 2021; GEO accession GSE161290), in which endothelial and mural cells were FACS-sorted from the dura of WT mice and profiled on the 10X Genomics Chromium platform. Filtered feature-barcode matrices for four samples were downloaded from GEO and imported into R using the ReadMtx function, and a Seurat object was created for each sample with CreateSeuratObject, retaining genes detected in at least 3 cells (min.cells = 3) and cells with at least 200 detected genes (min.features = 200). Sample and sex (male/female) identifiers were added as metadata, and the four objects were combined into a single object with merge. The percentage of mitochondrial reads was calculated per cell (PercentageFeatureSet, pattern = “^mt-”), and low-quality cells were removed by retaining only cells with >200 and <6,000 detected genes (nFeature_RNA) and <10% mitochondrial content. Counts were log-normalized (NormalizeData), the 2,000 most variable genes were identified (FindVariableFeatures), and expression values were scaled across genes (ScaleData) prior to principal component analysis (RunPCA). The first 15 principal components were used for shared-nearest-neighbor graph construction (FindNeighbors, dims = 1:15), Louvain clustering (FindClusters, resolution = 0.2), and uniform manifold approximation and projection (RunUMAP, dims = 1:15). Endothelial populations were identified by canonical marker expression (*Pecam1*, *Cdh5*, *Lyve1*, *Prox1*, *Flt4*, *Pdpn*, and *Ccl21a*), and the endothelial clusters were subsetted (subset) and reprocessed independently: cells were renormalized (NormalizeData), variable genes reidentified (FindVariableFeatures), scaled (ScaleData), and subjected to principal component analysis (RunPCA), followed by shared-nearest-neighbor graph construction (FindNeighbors, dims = 1:10), clustering (FindClusters, resolution = 0.1), and UMAP embedding (RunUMAP, dims = 1:10). Clusters were annotated as blood endothelial cells (*Pecam1*^*+*^, *Cdh5*^*+*^, *Sox17*^*+*^, and *CD34*^*+*^) or LECs (*Prox1*^*+*^, *Lyve1*^+^, *Flt4*^+^, *Mmrn1*^+^, *Pdpn*^+^, *Ccl21a*^+^, *Pecam1*^+^, and *Cdh5*^+^). Expression of the TNF receptors *Tnfrsf1a* and *Tnfrsf1b* was then examined within the LEC cluster.

### Statistical analysis

Two-tailed unpaired Student’s *t* tests were used for pairwise comparisons between two groups. For pairwise normalized comparisons between two groups, the Wilcoxon matched-pairs signed-rank test was used. One-way ANOVA, accompanied by appropriate multiple-comparison tests (as specified in the figure legends), was utilized to assess differences among three distinct groups. For comparisons of multiple factors, two-way ANOVA with appropriate multiple-comparisons tests (stated in figure captions) was used. Data were always presented as mean ± SEM. All statistical tests were conducted using GraphPad Prism (GraphPad Software, version 9.0.2). Heat-map visualization of protein log_2_-ratios was performed with heatmap (pheatmap: Pretty Heatmaps. R package version 1.0.12). P values were annotated as follows: NS P > 0.05, *P < 0.05, **P < 0.01, ***P < 0.001, and ****P < 0.0001.

### Online supplemental material

Fig. S1 provides validation of the α-syn PFF preparations, flow cytometry gating strategies and immune profiling following acute CSF α-syn exposure, analyses of fluorescent α-syn distribution and uptake, and assessment of glymphatic influx and OVA uptake by dural cell populations. Fig. S2 provides validation of the AAV9 α-syn constructs, characterization of α-syn accumulation in the dura following chronic CSF exposure, assessment of lymphatic drainage, and analyses of brain parenchymal pathology. Fig. S3 provides behavioral analyses of AAV9-GFP and AAV9-α-syn mice, reanalysis of scRNA-seq data to assess TNF receptor expression in dural LECs, and additional analyses of NF-κB activation, VE-cadherin phosphorylation, cytotoxicity, and TNF release in iLECs. Table S1 lists the human postmortem cases included in the study. Table S2 lists the antibodies used for immunocytochemistry, immunohistochemistry, and flow cytometry. Table S3 lists the iPSC lines used for LEC differentiation. Table S4 lists the media compositions used for iPSC culture and LEC differentiation.

## Supplementary Material

Table S1 shows human postmortem cases.

Table S2 shows the list of antibodies used.

Table S3 shows iPSC lines used for LEC differentiation.

Table S4 shows media compositions used for LEC differentiation.

## Data Availability

All data needed to evaluate the conclusions in the paper are present in the paper and/or the supplementary materials. The data underlying Fig. 4 F and Fig. S3, J–M are openly available in the Gene Expression Omnibus under the accession numbers GSE178498 and GSE161290, respectively.
