# Supplementary material for: Cerebrospinal fluid and dural accumulation of α-synuclein impairs CNS clearance in Parkinson’s disease
Source: J Exp Med. 2026 Oct 20;223(11):e20251245. doi: 10.1084/jem.20251245 (PMC13682444; doi:10.1084/jem.20251245)
Supplement: Supplementary file 1 [file jem_20251245_tables1.docx]

**Table S1: Human post-mortem cases**

| **Case** | **Age (years)** | **Sex (M/F)** | **Post-mortem delay (hrs)** | **Cause of death** |
| --- | --- | --- | --- | --- |
| **Control cases** | | | | |
| H243 | 77 | M | 13 | Ischemic heart disease |
| H246 | 89 | M | 17 | Myocardial infarction |
| H264 | 56 | M | 37 | Pancreatic adenocarcinoma |
| H268 | 90 | F | 25 | Enterococcus coli bacteraemia |
| H274 | 85 | M | 34 | Pneumonia |
| **Alzheimer’s cases** | | | | |
| AZ153 | 90 | F | 20 | Inanition |
| AZ159 | 90 | F | 7 | Respiratory arrest |
| **Parkinson’s disease cases** | | | | |
| PD65 | 67 | M | 2.25 | Parkinson’s disease |
| PD67 | 65 | M | 17 | Pneumonia |
| PD60 | 80 | M | 18 | Urosepsis |
| PD69 | 84 | F | 22 | Bronchoneumonia |
| PD79 | 77 | M | 6.5 | Parkinson’s disease |
| PD71 | 80 | M | 5.5 | Parkinson’s disease |
| PD57 | 84 | F | 22 | End-stage Parkinson’s disease |
| PD113 | 77 | F | 23 | Aspiration episode |
| PD114 | 80 | M | 16.5 | Chest infection |
| PD116 | 75 | M | 18 | LB dementia with PD plus syndrome |
| PD118 | 84 | M | 5.5 | Sepsis |
| PD123 | 69 | M | 21 | Cardio-respiratory arrest |
| PD130 | 82 | F | 25 | Inanition secondary to severe frailty and debility |
| PD132 | 80 | M | 13 | End stage Parkinson’s disease |
