# Supplementary material for: Cerebrospinal fluid and dural accumulation of α-synuclein impairs CNS clearance in Parkinson’s disease
Source: J Exp Med. 2026 Oct 20;223(11):e20251245. doi: 10.1084/jem.20251245 (PMC13682444; doi:10.1084/jem.20251245)
Supplement: Supplementary file 2 [file jem_20251245_tables2.docx]

**Table S2: List of antibodies used**

| Antigen | Species/Conjugate | Company | Cat. No. | ICC | IHC | FC |
| --- | --- | --- | --- | --- | --- | --- |
| CD3e-eF660 | Rat | ThermoFisher | 50-0032-82 |  |  | 1:200 |
| CD4-FITC | Rat | ThermoFisher | 11-0041-82 |  |  | 1:200 |
| CD8a-APC-eF780 | Rat | ThermoFisher | 47-008-82 |  |  | 1:200 |
| CD8-AF647 | Rat | ThermoFisher | MCD0821 |  |  | 1:200 |
| CD11b-PE-Cy7 | Rat | ThermoFisher | 25-0112-82 |  |  | 1:200 |
| CD11c-BV605 | Armenian Hamster | BD Biosciences | 563057 |  |  | 1:200 |
| CD16/32 | Rat | Biolegend | 101301 |  |  | 1:200 |
| CD19-BV480 | Rat | BD Biosciences | 566107 |  |  | 1:200 |
| CD31--PerCP-Cy5.5 | Mouse | Biolegend | 303132 |  |  | 1:200 |
| CD64-APC | Mouse | Biolegend | 139306 |  |  | 1:200 |
| CD45 | Rat | ThermoFisher | 564105 |  |  | 1:200 |
| CD45-PerCP-Cy5.5 | Mouse | BD Bioscience | 45-0451-82 |  |  | 1:62 |
| Anti-goat AF647 | Donkey | ThermoFisher | A21447 | 1:500 |  |  |
| Anti-mouse AF488 | Donkey | ThermoFisher | A21202 | 1:500 |  |  |
| Anti-mouse AF647 | Donkey | ThermoFisher | A31571 | 1:500 | 1:500 |  |
| Anti-rabbit AF488 | Donkey | ThermoFisher | A21206 | 1:500 |  |  |
| Anti-rabbit AF594 | Donkey | ThermoFisher | A21207 |  | 1:500 |  |
| Anti-rat AF594 | Donkey | ThermoFisher | A21209 |  | 1:500 |  |
| DPP4 | Mouse | R & D Systems | AF954 |  | 1:200 |  |
| F4/80-AF700 | Rat | ThermoFisher | 56-4801-82 |  |  | 1:200 |
| HLA-DR | Mouse | DAKO | MO775 | 1:500 |  |  |
| IBA1 | Goat | Abcam | ab5076 |  | 1:200 |  |
| GFAP | Rabbit | DAKO | Z0334 |  | 1:500 |  |
| Ly6C-BV421 | Rat | BioLegend | 128031 |  |  | 1:200 |
| Ly6g-PE | Rat | ThermoFisher | 12-9668-82 |  |  | 1:200 |
| LYVE1-eF660 | Rat- | ThermoFisher | 50-0443-82 |  | 1:200 |  |
| LYVE1-AF488 | Rabbit | Novus Biologicals | 50-0443-82 | 1:20 |  | 1:125 |
| MHCII-PE-Cy5 | Rat | ThermoFisher | 15-5321-81 |  |  | 1:2500 |
| NK1.1-BV510 | Mouse | BioLegend | 108737 |  |  | 1:200 |
| PDGFRβ | Goat | R&D Systems | AF385 | 1:1,000 |  |  |
| PROX1 | Rabbit | Abcam | Ab199359 | 1:100 |  |  |
| PU.1 | Rabbit | Cell Signalling | 2258 | 1:500 |  |  |
| VE-Cadherin | Goat | R & D Systems | AF938 | 1:500 |  |  |
| VE-Cadherin-PE-Cy7 | Rat | Biolegend | 138016 |  |  | 1:200 |
| VEGFR3 | Mouse | R & D Systems | MAB3491 | 1:100 |  |  |
| VEGFR3-PE | PE | Biolegend | 356204 |  |  | 1:200 |
| α-synuclein | Mouse | abcam | Ab1903 | 1:1000 | 1:1000 |  |
| α-synuclein (phospho s129) | Rabbit | Abcam | Ab59264-10 | 1:1000 |  |  |
| TNF | Rabbit | Abcam | Ab183218 |  | 1:2000 |  |
| α-synuclein | Rabbit | Abcam | Ab212184 | 1:1000 | 1:1000 |  |
| CD45 | Mouse | Abcam | Ab8216 | 1:500 |  |  |
| TCRβ-PE/Dazzle 594 | Mouse | Biolegend | 109239 |  |  | 1:200 |
| CD117-FITC | Rat | Biolegend | 105805 |  |  | 1:200 |
| SCA1-AF647 | Rat | Biolgend | 108118 |  |  | 1:200 |
| Lin-PB | Rat | Biolegend | 133310 |  |  | 1:200 |
| Ki67-BV605 | Rat | Biolegend | 652413 |  |  | 1:200 |
| CD135-PE-Cy5 | Rat | Biolegend | 135311 |  |  | 1:200 |
| CD150-BV650 | Rat | Biolegend | 115932 |  |  | 1:200 |
| CD48-BV750 | Hamster | Biolegend | 103449 |  |  | 1:200 |
| Hoechst 33342 |  | Molecular Probes | H1399 | 1:500 | 1:10000 |  |
| NIR Live/Dead-633 |  | Invitrogen | L10119A |  |  | 1:500 |
| TMEM119 | Rabbit | Abcam | Ab209064 |  | 1:500 |  |
| CD68 | Mouse | Invitrogen | MA5-13324 |  | 1:500 |  |
| Phospho-Ve-cadherin (Tyr658) | Rabbit | Invitrogen | 44-1144G | 1:500 |  |  |
| Podocalyxin | Goat | R&D Systems | AF1658 |  | 1:500 |  |
| α-SMA | Rabbit | Abcam | Ab5694 |  | 1:500 |  |
| NFκB | Rabbit | Abcam | Ab32536 | 1:500 |  |  |
| Tyrosine hydroxalase | Mouse | Santa Cruz | sc-25269 |  | 1:200 |  |
