# Supplementary material for: Cerebrospinal fluid and dural accumulation of α-synuclein impairs CNS clearance in Parkinson’s disease
Source: J Exp Med. 2026 Oct 20;223(11):e20251245. doi: 10.1084/jem.20251245 (PMC13682444; doi:10.1084/jem.20251245)
Supplement: Supplementary file 3 [file jem_20251245_tables3.docx]

**Table S3: iPSC lines used for LEC differentiation**

| iPSC Line | Source | Sex | Age | Ethnicity | Alias |
| --- | --- | --- | --- | --- | --- |
| ATCC-BYS0112 | ATCC | M | 31 | Non-Hispanic Caucasian | Line 1 |
| ATCC-BXS0116 | ATCC | F | 31 | Non-Hispanic Caucasian | Line 2 |
| KOLF2.1J | Jackson Laboratory | M | 55-59 | Non-Hispanic Caucasian | Line 3 |
