# Supplementary material for: Cerebrospinal fluid and dural accumulation of α-synuclein impairs CNS clearance in Parkinson’s disease
Source: J Exp Med. 2026 Oct 20;223(11):e20251245. doi: 10.1084/jem.20251245 (PMC13682444; doi:10.1084/jem.20251245)
Supplement: Supplementary file 4 [file jem_20251245_tables4.docx]

**Table S4: Media compositions used for LEC differentiation**

| Reagent | Stock Concentration | Final concentration/volume |
| --- | --- | --- |
| iPSC growth media | | |
| StemFlex | 1X | 1X |
| iPSC growth media—prior to mesoderm induction | | |
| StemFlex | 1X | 1X |
| Y-27632 (Dihydrochloride) | 10 mM | 10 µM |
| Mesoderm induction media | | |
| DMEM/F12 | 1X | 0.5X |
| Neurobasal | 1X | 0.5X |
| Glutamax | 100X | 1X |
| N2 | 50X | 1X |
| B27 | 50X | 1X |
| CHIR99021 | 3 mM | 8 µM |
| BMP4 | 100 µg/mL | 25 ng/mL |
| Lymphatic endothelial cell induction media | | |
| StemPro34 | 1X | 1X |
| Glutamax | 100X | 1X |
| Forskolin | 2 mM | 2 µM |
| VEGF-C | 100 µg/mL | 100 ng/mL |
| WNT5b | 100 µg/mL | 100 ng/mL |
| VEGF-A | 100 µg/mL | 20 ng/mL |
| Ang1 | 100 µg/mL | 20 ng/mL |
| BMP4 | 100 µg/mL | 20 ng/mL |
| IL3 | 100 µg/mL | 20 ng/mL |
| Lymphatic endothelial cell growth media | | |
| Endothelial Cell Medium | 1X | 1X |
| VEGF-C | 100 µg/mL | 100 ng/mL |
